# Advances in myocardial energy metabolism: metabolic remodelling in heart failure and beyond

**DOI:** 10.1093/cvr/cvae231

**Published:** 2024-10-25

**Authors:** Qiuyu Sun, Qutuba G Karwi, Nathan Wong, Gary D Lopaschuk

**Affiliations:** Cardiovascular Research Center, University of Alberta, Edmonton, AB T6G 2S2, Canada; Department of Pediatrics, University of Alberta, Edmonton, AB T6G 2S2, Canada; Division of BioMedical Sciences, Faculty of Medicine, Memorial University of Newfoundland, Saint John’s, NL A1B 3V6, Canada; Cardiovascular Research Center, University of Alberta, Edmonton, AB T6G 2S2, Canada; Department of Pediatrics, University of Alberta, Edmonton, AB T6G 2S2, Canada; Cardiovascular Research Center, University of Alberta, Edmonton, AB T6G 2S2, Canada; Department of Pediatrics, University of Alberta, Edmonton, AB T6G 2S2, Canada

**Keywords:** HFpEF, HFrEF, Fatty acid oxidation, Glucose oxidation, Ketone oxidation

## Abstract

The very high energy demand of the heart is primarily met by adenosine triphosphate (ATP) production from mitochondrial oxidative phosphorylation, with glycolysis providing a smaller amount of ATP production. This ATP production is markedly altered in heart failure, primarily due to a decrease in mitochondrial oxidative metabolism. Although an increase in glycolytic ATP production partly compensates for the decrease in mitochondrial ATP production, the failing heart faces an energy deficit that contributes to the severity of contractile dysfunction. The relative contribution of the different fuels for mitochondrial ATP production dramatically changes in the failing heart, which depends to a large extent on the type of heart failure. A common metabolic defect in all forms of heart failure [including heart failure with reduced ejection fraction (HFrEF), heart failure with preserved EF (HFpEF), and diabetic cardiomyopathies] is a decrease in mitochondrial oxidation of pyruvate originating from glucose (i.e. glucose oxidation). This decrease in glucose oxidation occurs regardless of whether glycolysis is increased, resulting in an uncoupling of glycolysis from glucose oxidation that can decrease cardiac efficiency. The mitochondrial oxidation of fatty acids by the heart increases or decreases, depending on the type of heart failure. For instance, in HFpEF and diabetic cardiomyopathies myocardial fatty acid oxidation increases, while in HFrEF myocardial fatty acid oxidation either decreases or remains unchanged. The oxidation of ketones (which provides the failing heart with an important energy source) also differs depending on the type of heart failure, being increased in HFrEF, and decreased in HFpEF and diabetic cardiomyopathies. The alterations in mitochondrial oxidative metabolism and glycolysis in the failing heart are due to transcriptional changes in key enzymes involved in the metabolic pathways, as well as alterations in redox state, metabolic signalling and post-translational epigenetic changes in energy metabolic enzymes. Of importance, targeting the mitochondrial energy metabolic pathways has emerged as a novel therapeutic approach to improving cardiac function and cardiac efficiency in the failing heart.

## 1. Introduction

Heart failure is a serious clinical syndrome that results in the inability of the heart to adequately pump enough blood to meet the body's needs for nutrients and oxygen. This results in heart failure patients having significant disabilities and a high mortality rate.^1^ Heart failure is often classified by the degree by which alterations in ejection fraction (EF) occur, including heart failure with reduced EF (HFrEF), heart failure with mildly reduced EF (HFmrEF), and heart failure with preserved EF (HFpEF).^2–4^ Heart failure is associated with multiple different co-morbidities, with coronary artery disease, hypertension, diabetes, obesity, valvular disease, arrhythmias, aging, and chronic kidney disease being prominent contributors to heart failure development. It is also becoming clear that alterations in energy metabolism also characterize the failing heart, resulting in an ‘energy deficit’ that contributes to heart failure severity.^5^ This compromised energy production results from a number of factors, which include impaired mitochondrial metabolism, alterations in energy substrate preference by the heart, and a decrease in cardiac efficiency.^6^

To sustain contractile function the heart must produce a large quantity of energy, in the form of adenosine triphosphate (ATP). There are no significant ATP reserves in the heart, and if not continually replaced, the heart would run out of ATP in 2–10 s, resulting in contractile failure.^6^ As a result, the continuous production of ATP must occur to maintain cardiac function. The heart normally achieves this by metabolizing a variety of fuels, primarily by mitochondrial oxidative phosphorylation (OXPHOS) and, to a lesser extent, from glycolysis. Any disruptions in these metabolic pathways that produce ATP can have catastrophic consequences on heart function. As a result, compromised cardiac energy production is an important contributor to heart failure severity.^7–11^

Despite the critical importance of energy production in maintaining heart function, what energy metabolic changes occur in heart failure it is still not completely understood. This is in part due to the many co-morbidities associated with heart failure. For instance, heart failure associated with diabetes or obesity can have very different effects on the use of fatty acids as an energy substrate by the heart, compared to heart failure associated with hypertension or coronary artery disease.^12–16^ The goal of this review is to overview what cardiac energetic changes occur in the various forms of heart failure. We will also discuss how optimizing energy metabolism may be used as an approach to prevent or treat heart failure.

## 2. Energy metabolism in the normal heart

**Figure 1:**
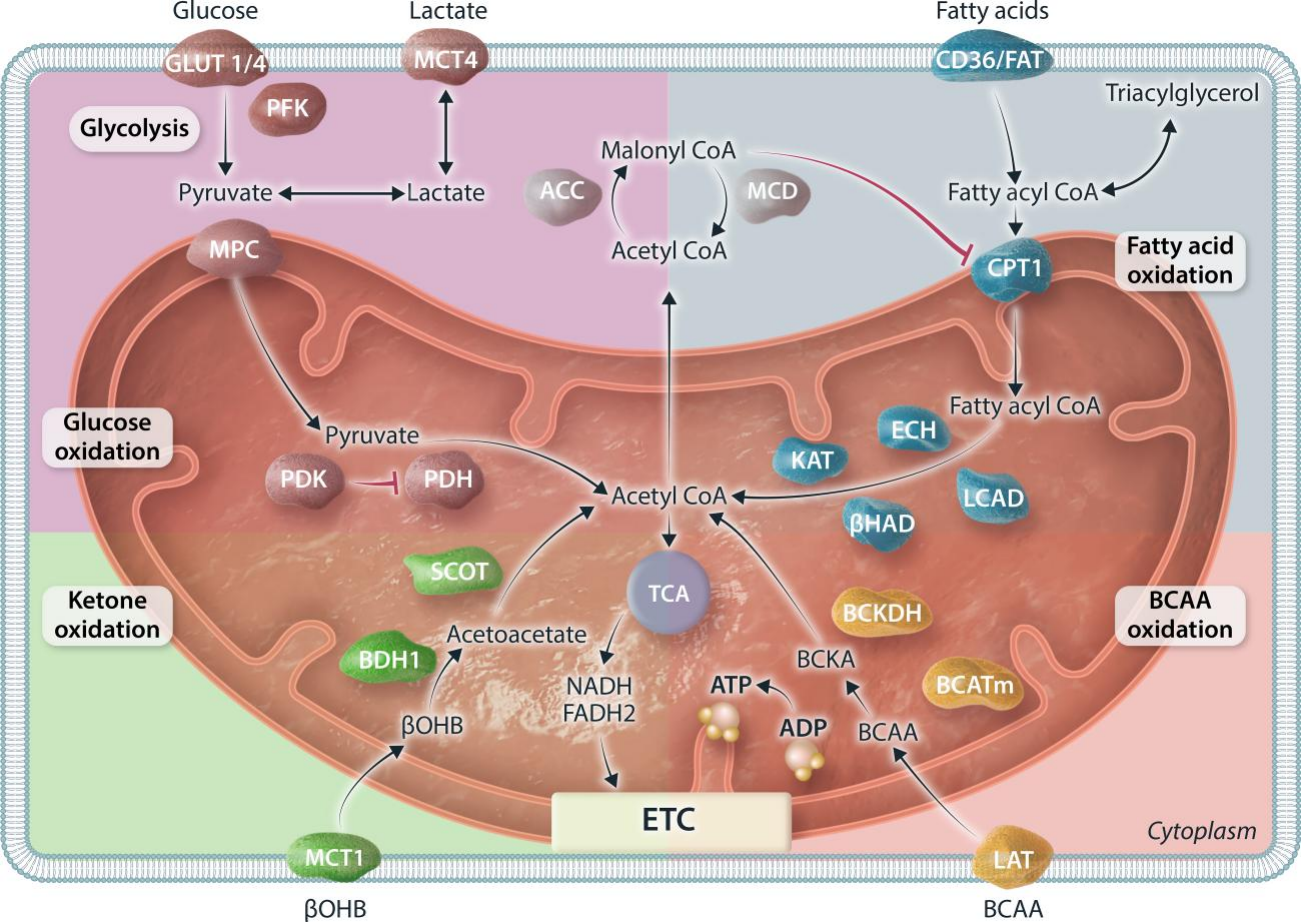
Depiction of major metabolic pathways and its associated key enzymes involved in metabolism of glucose, fatty acids, ketones, and BCAA in the heart. Glucose metabolism can be divided as glycolysis and downstream pyruvate metabolism/glucose oxidation. Fatty acid oxidation can be regulated by the endogenous inhibitor malonyl-CoA. Ketone oxidation is depicted using the major form of ketone bodies, βOHB. BCAA oxidation includes oxidation of leucine, isoleucine, and valine. All four metabolic substrates are converted to acetyl-CoA for entering the TCA cycle to generate reduced equivalent NADH and FADH2, which will enter the ETC and generate a proton gradient across the mitochondrial membrane to drive the action of ATP synthase for ATP genesis. GLUT1/4, glucose transporter 1/4; MCT4, monocarboxylate transporter 4; PFK, phosphofructokinase; MPC, mitochondrial pyruvate carrier; PDH, pyruvate dehydrogenase; PDK, PDH kinase; ACC, acetyl-CoA carboxylase; MCD, malonyl-CoA decarboxylase; CD36/FAT, fatty acid transporter; CPT1, carnitine palmitoyltransferase 1; LCAD, long-chain acyl-CoA dehydrogenase; ECH, enoyl CoA hydratase; βHAD, 3-OH-acyl-CoA dehydrogenase; KAT, 3-ketoacyl-CoA thiolase; BCAA, branched-chain amino acids; BCATm, mitochondrial BCAA amimotransferase; BCKDH, branched-chain α-ketoacid dehydrogenase; LAT, L-type amino acid transporters; βOHB, β-hydroxybutyrate; BDH1, βOHB dehydrogenase 1; SCOT, succinyl-CoA:3-ketoacid CoA transferase; TCA, tricarboxylic acid; NADH, nicotinamide adenine dinucleotide; FADH2, flavin adenine dinucleotide; ETC, electron transport chain; ADP, adenosine diphosphate; ATP, adenosine triphosphate.

In a healthy state, the heart can utilize a variety of energy substrates including carbohydrates (glucose and lactate), fatty acids, ketones, and amino acids to generate ATP.^6,17^ This omnivorous characteristic is crucial for supporting the high ATP demand of the heart for contractile function and for maintaining the action of various ion pumps. Importantly, the healthy heart is metabolically flexible and can switch its preferences towards using different energy substrates depending on its environment, including changes in energy substrate availability, hormonal control, inotropic state, and the workload of the heart. For example, the heart can rapidly increase the rate of ATP synthesis by up-regulating both glucose and fatty acid metabolism during intense exercise to accommodate the increase in energy demand.^17–20^ Alternatively, during fasting or low-carbohydrate supply, the heart can rewire its metabolism to increase its reliance on fatty acid oxidation for ATP production.^21,22^ The majority of myocardial ATP production (∼95%) is derived from mitochondrial OXPHOS, with glycolysis generating the remaining ATP (∼5%).^23,24^ Fatty acid oxidation contributes approximately 40–60% of the reduced equivalents for OXPHOS), carbohydrate metabolism (glucose and lactate) contributes 20–40%, ketone oxidation 10–15%, and amino acid oxidation the remainder (<2%) (*Figure 1*).

### 2.1 Fatty acid metabolism

Fatty acids are the major fuel for the heart, with oxidation of fatty acids contributing 40–60% of cardiac ATP production. Fatty acids are supplied to the heart from the circulation either as free fatty acids bound to albumin, or as fatty acids hydrolyzed by lipoprotein lipase from chylomicron and very-LDL (VLDL) triacylglycerols (TAG).^25–27^ The heart can also derive fatty acids from internal TAG stores, which continually cycle fatty acids through these TAG stores.^28^ Circulating free fatty acids enter the cardiomyocyte via facilitated diffusion or by fatty acid transport protein (FATP) or fatty acid translocase (FAT/CD36).^25,29,30^ Around 90% the fatty acids within the cytosol are shuttled into mitochondria for further oxidation, while the remaining 10% can be stored as TAG as an energy source backup. Fatty acid oxidation itself occurs within the mitochondria, with several steps required to first ‘activate’ the fatty acids and transport them into the mitochondria.^31–33^ Cytosolic fatty acids are first esterified to fatty acyl-CoA (a process consuming two high-energy phosphate bonds as ATP is converted to AMP), followed by the transfer of the fatty acid moiety to carnitine via the action of carnitine palmitoyltransferase 1 (CPT1) to form fatty acyl carnitine. CPT1 residing on the outer mitochondrial membrane works collaboratively with carnitine acyltranslocase and carnitine palmitoyltransferase 2 (CPT2), residing on the inner mitochondrial member, to transfer fatty acylcarnitine into the mitochondrial matrix, where it is converted back to fatty acyl-CoA. These acyl-CoAs then undergo ß-oxidation to produce reduced equivalents (NADH and FADH_2_) for the electron transport chain (ETC), as well as acetyl-CoA for the tricarboxylic acid (TCA) cycle. The passage of acetyl-CoA through the TCA cycle produces further reduced equivalents for the ETC, as well as some ATP via guanosine triphosphate (GTP). The ETC then uses the reduced equivalents in the presence of oxygen to phosphorylate adenosine diphosphate to ATP (OXPHOS).

A key step regulating fatty acid oxidation in the heart occurs at the level of mitochondrial CPT1. Malonyl-CoA is an endogenous inhibitor of CPT1.^34–36^ Malonyl-CoA can be generated by acetyl-CoA carboxylase (ACC) from cytosolic acetyl-CoA.^37,38^ Alternatively, malonyl-CoA can be converted back to acetyl-CoA by malonyl-CoA decarboxylase (MCD).^39,40^ When the demand for ATP is low, acetyl groups can be transferred out of the mitochondria to increase cytoplasmic acetyl-CoA supply for ACC, leading to increased malonyl-CoA production.^41^ This creates a negative feedback loop that inhibits fatty acid uptake into the mitochondria for further oxidation. In contrast, a decrease in malonyl-CoA levels, due to activation of AMP-kinase (which phosphorylates and inhibits ACC) or to increased MCD activity, accelerates fatty acid oxidation.^28,39,42^

### 2.2 Glucose metabolism

Glucose is an important source of fuel for the heart.^6^ Cardiomyocytes primarily taken up glucose via both insulin-dependent glucose transporter 4 (GLUT4) and insulin-independent glucose transporter 1 (GLUT1).^43^ Upon entry, glucose can have many fates, including being directed towards, glycogen synthesis, the polyol pathway, the hexosamine biosynthetic pathway (HBP), galactosamine and mannose synthesis, one carbon metabolism, or the pentose phosphate pathway (PPP), although the majority glucose passes through glycolysis to be converted to pyruvate.^6^ The conversion of glucose to pyruvate produces two ATP, which importantly does not require oxygen, and as such can be up-regulated dramatically when mitochondrial OXPHOS decreases, such as during oxygen-deficient conditions like ischaemia. Most of the pyruvate from glycolysis is then either taken up by the mitochondria and oxidized or converted to lactate and transported out the cardiomyocyte.

In addition to glycolysis, a second important source of pyruvate originates from lactate, which, once entered the cardiomyocyte, is converted to pyruvate by lactate dehydrogenase. To undergo mitochondrial metabolism, this pyruvate is transported into the mitochondria via a mitochondrial pyruvate carrier (MPC).^44^ The majority of pyruvate is then oxidized to acetyl-CoA via pyruvate dehydrogenase (PDH). If this pyruvate originates from glucose, the process is termed ‘glucose oxidation’, and if the pyruvate originates from lactate it is termed ‘lactate oxidation’. Pyruvate can also be converted to oxaloacetate and malate via pyruvate carboxylase to replenish the TCA cycle as an anaplerotic substrate. Acetyl-CoA produced from glucose and lactate oxidation can feed into the TCA cycle, where it has a similar fate as acetyl-CoA originating from fatty acid oxidation.

Like fatty acid oxidation, glucose oxidation can be regulated at many stages. First and foremost, the action of PDH, the rate-limiting enzyme for glucose oxidation, is regulated by phosphorylation. PDH kinase (PDK) phosphorylates and inhibits PDH, whereas PDH phosphatase removes the phosphate group and activates PDH.^45^ For example, PDK is stimulated in response to increased acetyl-CoA/CoA and NADH/NAD^+^ ratios, which suppresses PDH, and therefore glucose oxidation.

### 2.3 Ketone metabolism

Ketones, which can be an important source of fuel for the heart,^46,47^ are normally produced by the liver during fasting and starvation when blood glucose level drops. Liver ketogenesis produces three forms of ketones: β-hydroxybutyrate (βOHB), acetoacetate, and acetone. βOHB is the predominant ketone used by the heart. Circulating ketones are transported into the cardiomyocyte through a monocarboxylate transporter (MCT1) and then shuttled into the mitochondria for further oxidation. βOHB is first oxidized to acetoacetate by βOHB dehydrogenase (BDH1), followed by conversion to acetoacetyl-CoA by succinyl-CoA:3 oxoacid-CoA transferase (SCOT). The end-product of ketone oxidation is acetyl-CoA, which has a similar fate as acetyl-CoA produced from fatty acid or glucose oxidation. In contrast to glucose and fatty acid oxidation, metabolism of ketones is not under much regulatory control. Therefore, the rate of myocardial ketone oxidation is highly dependent on its availability, which is directly linked with blood ketone levels,^48^ and to the transcriptional regulation of ketolytic enzymes. Although ketone oxidation is not a major source of ATP production in the heart of healthy non-fasting individuals (around 10–15% of total ATP produced), it can become a more important source of ATP production during fasting, or in some forms of heart failure (as will be discussed in the following section).

### 2.4 Amino acid metabolism

Besides being crucial building blocks of proteins, amino acids can also be oxidized as a source of ATP production for the heart. Oxidation of branched-chain amino acids (BCAAs), namely leucine, isoleucine, and valine, has received the most attention in the heart.^49^ In a healthy heart, BCAA oxidation has a minor contribution to overall myocardial ATP production, (~2%).^50^ However, BCAAs and metabolites of BCAA oxidation, particularly branched-chain ketoacids (BCKAs), do have an important role as signalling molecules that regulate the balance of fatty acid and glucose oxidation,^51,52^ and the hypertrophic process.^53,54^ For example, BCAAs can stimulate the mammalian target of rapamycin (mTOR) signalling pathway,^53–55^ while BCKAs can inhibit insulin stimulation of glucose oxidation.^51,52^ Increased systemic BCAA can also promote the development of insulin resistance.^56^ As such, even though BCAA oxidation is not a major source of fuel for the heart, it is critically involved in regulating the overall metabolic profile of the heart.

Two key enzymes regulating BCAA oxidation are mitochondrial branched-chain amino-transaminase (BCATm) and branched-chain α-keto acid dehydrogenase (BCKDH).^57^ BCATm converts BCAA to BCKA through transamination, followed by oxidative decarboxylation by BKCDH. Similar to PDH, BCKDH is inhibited by its kinase, BCKDH kinase (BDK). On the other hand, dephosphorylation by protein phosphatase 2Cm (PP2Cm) can reactivate BCKDH.

## 3. Energy metabolism in the failing heart

**Figure 2:**
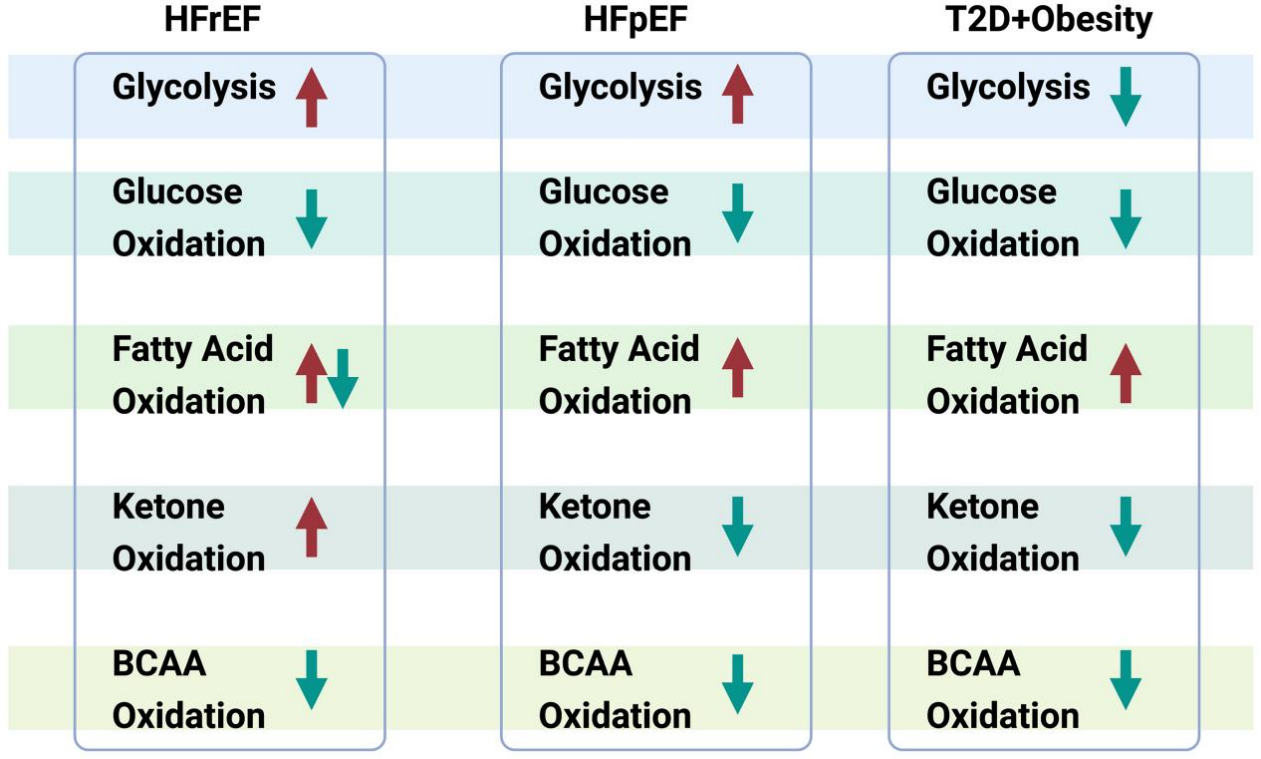
Alterations of myocardial energy metabolism in heart failure. Summary chart showing the alterations of five major energy metabolic pathways, including glycolysis, glucose oxidation, fatty acid oxidation, ketone oxidation, branched-chain amino acid (BCAA) oxidation in three different types of HFrEF, HFpEF, heart failure associated with obesity, and T2D. Arrow pointing upward indicates the corresponding pathway is stimulated, and arrow pointing downward indicates the corresponding pathway is suppressed.

The failing heart undergoes dramatic alterations in energy substrate utilization and overall metabolic profile, which can not only lead to a disrupted balance of cardiac energy metabolism but also to an overall diminished ATP production (*Figure 2*). The concept of the ‘energy-starved’ failing heart was first proposed in 1939 by Herrmann and Decherd,^58^ and has since been demonstrated in a number of studies.^5,59–64^ However, it should be clarified that the energy starvation concept can be misleading to think there is a lack of energy source, but in reality, the delivery of both oxygen and substrates to the heart is not limited. Therefore, another concept promoted by the Taegtmeyer group summarized it with the words ‘failure in the midst of plenty’.^65,66^ This energy deficiency/failure in midst of plenty occurs primarily due to a compromised mitochondrial oxidative metabolism and alterations in the source of ATP production.^50,62,67–69^ The failing heart has a decreased cardiac efficiency (cardiac work/O_2_ consumed).^69–72^ This decrease in cardiac efficiency is due, in part, to alterations in the source of fuel used by the heart. However, the precise alteration of each metabolic pathway in heart failure has yet to be universally established. This is particularly true for potential alterations in fatty acid oxidation in the failing heart, where many studies propose that cardiac fatty acid oxidation is decreased,^73–77^ while others suggest that fatty acid oxidation is either increased,^78–80^ or not changed.^81–83^ As will be discussed, these discrepant results may, at least in part, be explained by differences in disease aetiology, co-morbidities involved with heart failure, differences in transcriptional and post-translational control of energy metabolisms, and/or the severity of the disease.^84^

Fuel source in the failing heart is an important consideration, as the efficiency of ATP production can vary dramatically depending on the energy substrate used. This is important in the failing heart, which not only may have an ‘energy deficit’ but may also be oxygen limited. Although the oxidation of fatty acid produces a large amount of ATP per each molecule oxidized, it is the least efficient fuel in terms of ATP produced per molecule of oxygen consumed.^6^ Glucose is the most efficient energy substrate, with each molecule of glucose passing through glycolysis producing two ATP without the expense of oxygen. However, if glycolysis is uncoupled from glucose oxidation (i.e. the pyruvate from glycolysis is not taken up and oxidized by the mitochondria) it produces lactate and H^+^'s as metabolic by-products that can also contribute to a decrease in cardiac efficiency. Ketones have been proposed to be a ‘thrifty’ or ‘super fuel’ for the failing heart,^85,86^ but while they are a more efficient energy substrate than fatty acids, they are less efficient than glucose.^87^

### 3.1 Cardiac energy metabolism in HFrEF

HFrEF is characterized by a reduced EF (<40%), with ischaemic heart disease and hypertension being two important causes of HFrEF. HFrEF is associated with marked alterations in the overall capacity for cardiac energy production, as well as in alterations of cardiac fuel source.^6^ Although there is still a lack of consensus on the precise alterations of each metabolic pathway, it is well-accepted that the failing heart in HFrEF has impaired overall oxidative capacity and lowered myocardial ATP production (*Figure 2*).^5^ As discussed earlier, proper mitochondria function is pivotal for energy production, as OXPHOS occurs within mitochondria and provides around 95% of the total ATP produced. Both clinical and pre-clinical studies have shown that mitochondrial function is disrupted in HFrEF, which can explain the overall impaired cardiac energy production.

### 3.2 Alterations of glucose metabolism (glycolysis and glucose oxidation) in HFrEF

There is a widely held concept that the failing heart ‘switches from fatty acid to glucose metabolism’.^73,76,88,89^ However, we propose that this concept would be more fittingly re-worded as the failing heart ‘switches from mitochondrial OXPHOS to glycolysis to produce ATP’. An overall decrease in mitochondrial OXPHOS occurs in HFrEF, accompanied by an increase in glycolysis.^81,90–92^ However, cardiac glucose oxidation decreases in HFrEF in parallel to the decrease in mitochondrial OXPHOS.^83,93^ Therefore, it would be inaccurate to suggest that the failing heart is switching to glucose metabolism, given that glucose oxidation (which produces the majority of ATP originating from glucose), is markedly impaired.

Increased glycolytic ATP production in HFrEF may be an attempted compensatory response to a decrease in mitochondrial ATP production. Increased rates of cardiac glycolysis have been demonstrated in HFrEF and hypertrophic animal studies induced with transverse aortic constriction (TAC),^93^ or abdominal aortic constriction (ACC).^83,91^ This is further supported by human studies using metabolomics with heart samples collected from patients with HFrEF receiving left ventricular assist device.^92^ Diakos *et al.*^78,92^ found that glucose uptake and glycolytic rates were up-regulated in myocardial samples from patients with HFrEF compared to those of non-failing control. This increase in cardiac glycolytic rates, is also seen in animal models of HFrEF, such as in swine^94^ and rats.^91,95–97^

While glycolysis is up-regulated in the failing heart, the subsequent mitochondrial oxidation of glycolytically derived pyruvate (glucose oxidation) is actually decreased. This is supported by evidence from both preclinical studies using different animal models of HFrEF,^81,90,94–96^ and from human data collected from patients with HFrEF.^98^ While most studies support impaired cardiac glucose oxidation in the failing heart, opposing results showing that in canine model of pacing-induced heart failure, glucose oxidation is actually stimulated instead of being suppressed.^99^ This may have occurred due to the marked increase in glucose uptake and glycolysis in these hearts, as glucose uptake rates were 4 × higher in hearts from dogs with heart failure than that of control dogs, providing a large amount of pyruvate available for oxidation.

The decrease of glucose oxidation in HFrEF is tightly linked with the suppressed activity of PDH. This is evidenced by a study using hyperpolarized carbon-13 (^13^C) magnetic resonance spectroscopy to show that the activity of PDH diminished accordingly with the progression of cardiac dysfunction after myocardial infarction (MI) surgery in rats.^96^ Similar results were also shown in rapid pacing-induced heart failure in pigs, where the flux of PDH was reduced as evidenced by a 67% reduction in the [^13^C] bicarbonate/[1-^13^C] pyruvate ratio.^94^ Of importance, this suppression of cardiac glucose oxidation rates can, at least in part, contribute to the pathogenesis of HFrEF. Cardiac-specific knockout of PDH results in accelerated development of left ventricular hypertrophy and a marked impairment of systolic function, suggesting the imperative role of cardiac glucose oxidation in maintaining proper cardiac structure and function.^100^

Prior to PDH, another regulatory step of glucose oxidation is mitochondrial pyruvate uptake by MPC. MPC is located on the inner mitochondrial membrane and controls for pyruvate transfer into the mitochondria. In mice with a cardiac-specific MPC deletion, an age-dependent pathologic cardiac hypertrophy and LV dilation develop, ultimately leading to premature death.^101^ As expected, MPC deletion decreases cardiac glucose oxidation rates without any changing glycolytic rates.^101^ Importantly, this suppression of glucose oxidation occurs with increased fatty acid oxidation rates.^101^ This suggests that maintaining myocardial glucose oxidation is central to cardiac energetics and overall heart function. Another study shows that overexpression of MPC in cultured H9c2 cardiomyocytes attenuates drug induced cellular hypertrophy, suggesting that stimulating cardiac glucose oxidation is beneficial in ameliorating adverse remoulding.^102^ In the clinical setting, MPC expression is down-regulated in heart samples collected from patients with chronic heart failure.^102^ Therefore, this suggests that impaired myocardial glucose oxidation with MPC deficiency can, at least in part, contribute to the development of cardiac hypertrophy and the progression into heart failure.^101^

A recent study by Murashige *et al.*^50^ assessed myocardial uptake of major energy substrates in hearts from patients with HFrEF and HFpEF compared to healthy control participants. The authors found that around 85% of myocardial ATP is attributed to fatty acid sources, 6.4% from ketone bodies, 4.6% from amino acids, and 2.8% from lactate in hearts from patients with HFpEF.^50^ On the contrary, these relative substrate contributions to overall cardiac ATP production differs greatly in the hearts of patients with HFrEF, where fatty acids accounted for 71%, ketones for 16.4%, amino acids for 6.7%, and lactate for 5%.^50^ Curiously, the study did not report any net glucose uptake by the heart. This contrasts many earlier studies, including the original study from 1954 by Richard Bing *et al.*^103^ which showed significant glucose extraction by the heart. Other studies used metabolomic assessment with myocardial biopsies from patient with end-stage heart failure also found an accumulation of pyruvate and glycolytic intermediates,^92,102,104,105^ suggesting overall impaired pyruvate metabolism/glucose oxidation by the heart, yet the heart is actively using glucose as an energy substrate, but with the pyruvate metabolism step being suppressed.

In summary, the dysregulated glucose metabolism observed in HFrEF is manifested as an increase of glycolysis coupled with a suppression of glucose oxidation. This uncoupling between the two processes can lead to the accumulation of lactate and protons, contributing to cellular acidosis. These protons can impair cardiac function by interfering with calcium binding to contractile proteins and inhibiting slow calcium currents.^41,106,107^ Disrupted ionic balance within the cardiomyocytes also requires ATP to support the action of various ionic pumps to re-establish ionic homeostasis, which can decrease cardiac efficiency, as ATP is directed towards to regain ionic balance instead of supporting contractile function. Interestingly, this uncoupling between glycolysis and glucose oxidation has been recognized as a potential therapeutic target. In support of this, pharmacological stimulation of cardiac glucose oxidation has been shown to improve cardiac function and lower lactate production in a rat model of congestive heart failure.^75^

### 3.3 Alterations of fatty acid oxidation in HFrEF

Cardiac fatty acid oxidation also undergoes significant alterations in the setting of HFrEF. However, the precise pattern of these changes remains controversial, with studies proposing increased,^78^ decreased,^88,99,108^ or no change^81,82^ in cardiac fatty acid oxidation in HFrEF. While the idea that fatty acid oxidations are impaired in HFrEF has been proposed and accepted in general,^64,109^ most evidence is based on a decreased transcription of enzymes involved in fatty acid oxidation,^88,110^ as opposed to direct flux measurements of fatty acid oxidation. In the following section, we will critically discuss the major findings from both pre-clinical and clinical studies.

In the clinical setting, it has been repeatedly shown that circulating fatty acid levels are elevated in patients with heart failure.^111,112^ Related to this, the rate of cardiac fatty acid oxidation and fatty acid uptake is negatively correlated with the degree of insulin resistance and left ventricular function.^113^ Increasing the availability of circulating free fatty acids results in greater fatty acid uptake and oxidation by the heart in depressed cardiac function and an intense insulin resistant state.^114^ Additionally, this result is further supported by a study using positron emission tomography (PET) imaging techniques, showing that patients with severe depression of cardiac function (% EF around 25%) had a significant increase in fatty acid uptake rates compared to healthy controls.^78^ However, there have been some opposing results that found decreased fatty acid uptake in patients with non-ischaemic heart disease,^77^ and with idiopathic dilated cardiomyopathy.^76^ These divergent results might be partially due to the differences in disease aetiology, where patients assessed across various studies might have different degree of EF and/or involvement of other risk factors, such as obesity and/or metabolic syndromes.

The findings from preclinical studies also provide conflicting results. Using TAC models with isolated working heart perfusions, several studies have shown that the absolute rates of fatty acid oxidation are not significantly altered.^115,116^ However, it should be recognized that cardiac work is an important determinant of oxidative rates, and cardiac work is decreased in the TAC hearts.^90,115,116^ This would significantly increase fatty acid oxidation rates when normalized to per unit work.^90^ On the contrary, other studies have shown that cardiac fatty acid oxidation is impaired in rats,^108^ and canines^117^ with heart failure. Although the absolute rates of cardiac fatty acid oxidation were shown to be unchanged or decreased in these studies, the relative percentages of fatty acid contributing to overall myocardial ATP production are, in fact, elevated.^118^

Fatty acid metabolism is complicated in nature, as it is regulated at multiple levels.^118^ One of which is through the action of insulin. Insulin can not only positively regulate glucose metabolism but also negatively regulate fatty acid metabolism.^119^ In a healthy heart, insulin can readily inhibit the rate of fatty acid oxidation.^120,121^ However, the failing heart becomes insulin resistant, as evidenced by the lack of fatty acid oxidation inhibition with insulin.^90,120,121^ Importantly, a recent study by Watson *et al.*^122^ found that in patients with non-ischemic HFrEF, the heart was preserved with metabolic flexibility. Metabolic rates of glucose, fatty acids, and ketones were dependent on availability of substrates infused, being whether glucose or intralipids. Therefore, it is important to note that metabolism of fatty acids can be varied with respect alterations of the circulating levels.

### 3.4 Alterations of ketone oxidation in HFrEF

Recent interest has focused on ketones as a fuel source for the failing heart. Since the failing heart is energy-starved/failure in midst of plenty, ketone oxidation may provide the heart with an additional energy source. In patients with heart failure^123^ and in mice with heart failure,^124^ blood ketone levels are elevated. Two important studies by Bedi *et al.*^125^ and Aubert *et al.*^124^ examining metabolomics and ketolytic protein expression in humans and mice suggested that cardiac ketone oxidation is increased in HFrEF. Direct flux measurements by us in isolated working hearts from HFrEF mice also showed that ketone oxidation rates increase in the failing heart.^90^ A study by Voros *et al.*^80^ also showed increased myocardial uptake of βOHB and acetoacetate in patients with HFrEF compared to healthy controls. This increase in ketone oxidation appears to represent an adaptive mechanism to protect the failing heart.^126^ Support for ketone oxidation in the failing heart being adaptive also originates from studies in which genetic overexpression^127^ or knockout (KO)^126^ of genes involved in ketone oxidation were performed. Cardiac-specific deletion of BDH1 worsens cardiac remodelling and further impairs cardiac function in mice subjected to TAC/MI-induced heart failure.^126^ In contrast, overexpressing BDH1 decreases cardiac fibrosis and improves contractile function in mice subjected to TAC-induced heart failure.^127^ While it is has been proposed that ketones can act as a thrifty fuel for the failing heart,^85^ we demonstrated that increasing cardiac ketone oxidation does not increase cardiac efficiency.^48^ Similarly, in HFrEF patients with decreased cardiac efficiency, acutely increasing ketone supply to the heart improves heart function but does not improve cardiac efficiency.^70^ We propose that the major benefit of increasing cardiac ketone oxidation is to provide the heart with an additional source of fuel to increase ATP production to the energy-starved/failure in midst of plenty failing heart.^128^

### 3.5 Alterations of BCAA oxidation in HFrEF

BCAA, namely leucine, isoleucine, and valine, are important amino acids for both systemic and cardiac energy metabolism, as well as for assisting protein synthesis.^129^ Interestingly, recent studies have proposed that impaired BCAA metabolism correlates with the degree of insulin resistance and cardiac dysfunction in HFrEF, as evidenced by elevated circulating levels of BCAAs and its metabolites BCKAs.^52,130,131^ While elevated plasma levels of BCAA can potentially translate to an increased metabolism of BCAA for myocardial ATP production,^129^ it is important to recognize that BCAA oxidation accounts for less than 2% of overall ATP production.^132^ It is also apparent that cardiac BCAA oxidation is impaired in HFrEF,^130,131,133^ although not all studies support this.^134^ This appears to be due to phosphorylation and inhibition of BCKDH, resulting in an increase in both cardiac BCAAs and BCKAs.^131,135,136^ Despite having a minor contribution to cardiac ATP production, BCAAs and BCKAs may be important signalling molecules that adversely affect cardiac structure and energy metabolism in HFrEF.^132^ Genetic deletion of cardiac BCATm, the first enzyme of BCAA oxidation that converts BCAA to BCKA, leads to a build-up of BCAAs in the heart, which can activate mTOR and promote hypertrophy.^132^ Interestingly, deletion of BCATm deceases BCKA levels, resulting in an improved cardiac insulin signalling and enhanced insulin-stimulated glucose oxidation.^132^ However, the beneficial effects of BCATm deletion on glucose oxidation rates in HFrEF hearts, are offset by adverse remodelling due to increased BCAA levels.^132^

## 4. Cardiac energy metabolism in metabolic phenotype of HFpEF

HFpEF is a specific type of heart failure characterized by diastolic dysfunction and heterogeneous pathophysiology.^137^ Patients with HFpEF are typically older and associated with diseases such as type 2 diabetes (T2D), obesity, and hypertension.^138^ The prevalence of HFpEF is increasing at an alarming rate,^139^ and is predicted to reach 8.5 million by 2030 for the US population alone, and a great proportion of that population (∼ 6 million) will be attributed to elderly people with aged over 65 years.^140^ Even more alarming is that there are still few clinically effective treatments for HFpEF. Most effective medications for HFrEF fail to show any benefits in HFpEF.^141^ As outlined by Sanjv *et al.*,^142^ such lack of promising results from clinical trials in HFpEF may be explained by the high phenotypic heterogeneity. Specifically for the metabolic phenotype of HFpEF, one potential therapeutic approach is to optimize cardiac energy metabolism. However, this first requires a better understanding of what energy metabolic changes occur in HFpEF hearts.

In a recent study by Murashige *et al.*,^50^ the authors systematically assessed the gradient differences of metabolites between the radial artery and coronary sinus to quantify the uptake of various metabolites of the hearts in patients with HFpEF and HFrEF. The study found that the contribution of ketones to overall myocardial ATP was 6.4% in patients with HFpEF, whereas this number almost tripled (16.4%) in patients with HFrEF. In line with this, another metabolomic study from Hahn *et al.*^143^ showed that both plasma and myocardial levels of ketones are higher in patients with HFrEF but not HFpEF.

Clarifying the energy metabolic changes in HFpEF is hindered by the lack of animal models that can fully characterize the complex pathophysiology of HFpEF. However, recent studies have introduced several animal models for studying HFpEF, such as the ‘2-Hit’,^144^ ‘3-Hit’,^145^ and many others.^146–148^ These animal models aim to recapitulate the human HFpEF phenotype with multiple co-morbidities. Although they might not be the ‘perfect’ models hitting all multitude of syndromes observed in patients,^149^ they are useful to investigate the underlying pathology of HFpEF. That being said, very few studies using these models have actually directly assessed cardiac energy metabolism in HFpEF (*Figure 2*).

A ‘2-Hit’ model of HFpEF has been described where mice received both a metabolic insult from a 60% high-fat diet (HFD) and a haemodynamic insult from the endothelial nitric oxide synthase (eNOS) inhibitor L-NAME to induce hypertension.^144^ This 2-Hit mouse model results in diastolic dysfunction, as evidenced by the elevated *E*/*E*′ ratio, with preserved EF.^144^ Importantly, a study from Tong *et al.*^150^ employed this HFpEF protocol in mice and found an impaired cardiac fatty acid oxidation associated with hyperacetylation of enzymes in the fatty acid oxidation pathway. The rate of fatty acid oxidation was measured using permeabilized mitochondria with 25 μM palmitoyl-carnitine to assess the oxygen consumption rates (OCR).^150^ Although not the focus of the study, these mitochondrial OCR studies also showed impaired pyruvate metabolic rates, suggesting an impaired cardiac glucose oxidation in HFpEF.^150^ This suppression of glucose oxidation was also observed in our recent study where we employed the ‘2-Hit’ HFpEF mouse model and directly measured cardiac energy metabolism in isolated working hearts. Of importance, insulin-stimulated glucose oxidation rates are significantly decreased in HFpEF hearts.^121^ Contrary to Tong *et al.*,^121^ however, we found that cardiac fatty acid oxidation is not decreased but rather increased in HFpEF hearts. Because the loss of glucose oxidation is compensated by fatty acid oxidation, this results in overall preserved ATP production rates.^121^ As such, the heart in HFpEF is energetically preserved, instead of being energetically starved/failure in midst of plenty as is seen in HFrEF.^90^ We believe, this is largely because the heart is switching from oxidation of glucose to fatty acid in the setting of HFpEF, which is attributed to the impaired regulation of insulin on energy metabolism.^121^ At the molecular level, we observed an increased expression of PDK4 and associated phosphorylation of PDH, the rate-limiting enzyme of glucose oxidation. As a result, one possible avenue to restore the energy balance in the HFpEF heart is to stimulate cardiac glucose oxidation by inhibiting the action of PDK4. In support of this, we recently showed that in aged female mice subjected to the 2-Hit protocol, pharmacological inhibition of PDK4 restored glucose oxidation rates and improved cardiac function.^151^

In a ‘3-Hit’ model, Deng *et al.*^145^ induced HFpEF with long-term (13 months) high-fat feeding combined with deoxycorticosterone pivalate challenge. Interestingly, Deng *et al.* found that fatty acid oxidation decreased in these hearts, but unfortunately no assessment of glucose oxidation was made. Interestingly, ketone (ßOHB) oxidation was decreased by around 50% in HFpEF mice hearts assessed using carbon 13 nuclear magnetic resonance isotopomer analysis. This was associated with a lower expression of BDH1 enzymes. Similarly, genes for fatty acid uptake were also found to be down-regulated in ‘3-Hit’ HFpEF mouse hearts, but with only modest changes in the expression of β-oxidation genes. The decrease in ketone oxidation observed in the preclinical ‘3-Hit’ mouse heart was also found in human patients with HFpEF.^50^ Therefore, it is likely that the availability of that fuel source dictates the differential metabolism of ketones in HFpEF vs. HFrEF. That being said, fasting ketone levels are significantly higher in HFpEF mice,^145^ so another regulatory mechanism could potentially exist that also contributes to the lessening of cardiac ketone oxidation.

As discussed earlier in HFrEF section, increasing ketone supply to the heart may be beneficial as an extra fuel source for the failing heart. This approach has been tested in the setting of HFrEF with promising results. However, it remains unclear as whether this would be equivalently effective in the setting of HFpEF. Of relevance, SGLT2 inhibitors have recently been shown to lower hospitalization rates in patients with HFpEF.^152^ One of the main effects of SGLT2 inhibition is to induce a fasting mimetic state, therefore leading to endogenous ketogenesis and elevated plasma ketone levels,^153^ which might be one possible mechanism of SGLT2 inhibition, at least in part, in providing an extra source of fuel for the failing heart. However, direct flux measurements of ketone oxidation of the heart in the setting of HFpEF is still limited, and whether raising ketone availability to the heart, when the heart is already experiencing down-regulation of ketone metabolism, might be something beneficial or detrimental remains to be elucidated.

Interestingly, the severity of HFpEF in the ‘2-Hit’ model shows strong genetic and sex differences. Younger female mice are protected against the ‘2-Hit’ protocol, as evidenced by a smaller elevation in the *E*/*E*′ ratio and hypertrophy in female mice vs. male mice.^154^ However, it has not been determined if this is due to differences in cardiac energy metabolism. In contrast, Cao *et al.* suggested that female mice subjected to the HFpEF 2-Hit protocol develop a more severe diastolic dysfunction than male mice and linked this to differences in mitochondrial DNA (mtDNA) levels. Assessment of mitochondrial respiratory capacity showed a reduction in both basal and maximal respiration rates in mitochondrial from female compared to male hearts, as well as lower activity of Complex II and Complex IV of the ETC. In aging female mice subjected to the 2-Hit HFpEF protocol, we recently observed a marked increase in cardiac fatty acid oxidation and a decrease in insulin-stimulated glucose oxidation and ketone oxidation, similar to what we observed in younger male mice subjected to the 2-Hit protocol.^121^

Very little is known regarding what happens to cardiac amino acid metabolism in HFpEF. A clinical study from Hahn *et al.*^143^ found that BCAA levels are elevated in HFpEF myocardium, but metabolites of BCAA were lower, suggesting impaired BCAA oxidation. However, in this study, one of the major BCAA metabolites, BCKA was not measured. It is plausible that both BCKA and BCAA levels are elevated in HFpEF myocardium, as gene expression of enzymes that conve BCAA to BCKA, branched-chain amino acid transaminase 2 (BCAT2), is increased, in parallel with a decrease in downstream enzymes that catabolize BCKA. This is potentially important as BCKAs inhibit cardiac insulin-stimulated glucose oxidation,^132^ which is decreased in HFpEF.^121^

## 5. Cardiac energy metabolism in heart failure induced by obesity and T2D

Obesity and T2D are two prominent metabolic disorders that are closed related to heart failure progression.^155,156^ Obesity is a condition where the body accumulates excessive adipose tissue,^157^ whereas T2D is often characterized as a dysregulated glucose homeostasis.^158^ Both conditions are associated with insulin resistance.^159^ Insulin is an important regulator of energy metabolism and plays a key role in balancing glucose and fatty acid metabolism. In particular, obesity was proposed to be more strongly associated with risks of HFpEF than HFrEF.^160^ Similarly, another study showed that many patients with HFpEF also have T2D. Therefore, both obesity and T2D are highly prevalent in HFpEF,^161^ and are relevant for the progression of cardiac dysfunction.

In obesity, myocardial fatty acid oxidation rates are elevated.^162–165^ This is partially attributed to the higher circulating levels of free fatty acids in the bloodstream, so that and present themselves as readily available energy substrate to the heart (*Figure 2*). Many preclinical studies using isolated working heart perfusions have shown that myocardial fatty acid oxidation rates are elevated in obese mice.^121,166–168^ For example, in leptin-deficient *ob/ob* mice, obesity was progressively developed with concurrent development of insulin resistance and glucose intolerance.^164^ Systemic insulin resistance occurs in parallel with cardiac insulin resistance, where insulin stimulated glucose uptake and glucose oxidation is impaired at the level of the heart.^164,169^ Importantly, this suppression of glucose metabolism occurs simultanouesly with an increase in myocardial fatty acid oxidation.^121,164,166–168^ Overall, the heart in obesity switches from glucose oxidation towards fatty acid oxidation, becoming less metabolic flexible and less responsive to insulin.^121,164,166–168^ The paradoxical change of glucose and fatty acid metabolism^170^ is partly mediated through the inhibitory action of acetyl-CoA derived from fatty acid oxidation suppressing the activity of PDH and impairing glucose oxidation.^16^ Additionally, increased fatty acid oxidation flux will lead to more production of NADH and FADH_2_ from the TCA cycle, which would also negatively regulate PDH activity.^171^ Similar results were found by Buchanan *et al.*,^165^ showing that both *ob/ob* and *db/db* mice develop impaired glucose oxidation coupled with increased fatty acid oxidation as early as 4 weeks of age, which corresponds to the onset of systemic impaired glucose homeostasis and insulin resistance. This switch to fatty acid oxidation occurs with increased rates of myocardial oxygen consumption and lowered cardiac efficiency,^165^ as fatty acids are a less energy-efficient energy substrate compared to glucose.

Similar results were also found in the clinical setting.^162^ Peterson *et al.*^162^ reported that insulin resistance is closely related to high myocardial fatty acid uptake and oxidation in obese patients. Other studies have shown that patients with insulin resistance have increased lipolysis from adipose tissue,^172^ which would explain the higher circulating free fatty acids levels.

In diabetes, plasma glucose and fatty acids are also elevated, due to the combined effects of both stimulated hepatic glucose production and suppressed glucose uptake to peripheral tissue due to insulin resistance. Increased fatty acids levels in the bloodstream can be attributed to increased lipolysis of adipose tissues and elevated hepatic lipoprotein production. Yet, this increase in plasma substrate levels observed in diabetes is not directly correlated with substrate uptake rates in the heart.^173^ In particular, glucose uptake is controlled by GLUT1 and GLUT4. Insulin regulates the translocation of GLUT4 to sarcolemma membrane. In diabetes, GLUT4 translocation is impaired,^174^ which leads to a decrease of myocardial glucose uptake.^175^ Additionally, the expression of GLUT4 is also negatively regulated with increased intracellular levels of fatty acids. In fact, fatty acid uptake is increased in streptozotocin-induced diabetes.^176,177^ It is important to note that such increased uptake of fatty acids uptake might exceed mitochondrial oxidative capacity of metabolizing fatty acids, leading to incomplete ß-oxidation. This can cause an accumulation of unoxidized fatty acids, and possible conversion to harmful intracellular lipid intermediates, which suggest the danger of lipotoxicity.^178–181^

Since the heart switches from glucose oxidation to fatty acid oxidation in diabetes and obesity, it is reasonable to suggest that either inhibiting fatty acid oxidation or stimulating glucose oxidation may be a strategy to reconstruct the overall metabolic balance of the heart.^182,183^ Studies in a rat model of diabetic cardiomyopathy that is induced with streptozotocin injection showed that insulin resistance is improved with the PDK inhibitor, dichloroacetate (DCA), which leads to increased PDH flux, myocardial glucose oxidation, and overall improved cardiac function.^182^ On the other hand, treatment with a CPT1 inhibitor, oxfenicine, to suppress fatty acid oxidation also improves whole-body glucose tolerance and insulin sensitivity in mice fed with 12 weeks of HFD.^183^ Improved glucose homeostasis and clearance are also evidenced by increased expression of membrane GLUT4 and increased PDH activity in gastrocnemius skeletal muscle.^183^

## 6. Molecular mechanisms contributing to altered energy metabolism in heart failure

### 6.1 Alterations in mitochondrial function

**Figure 3:**
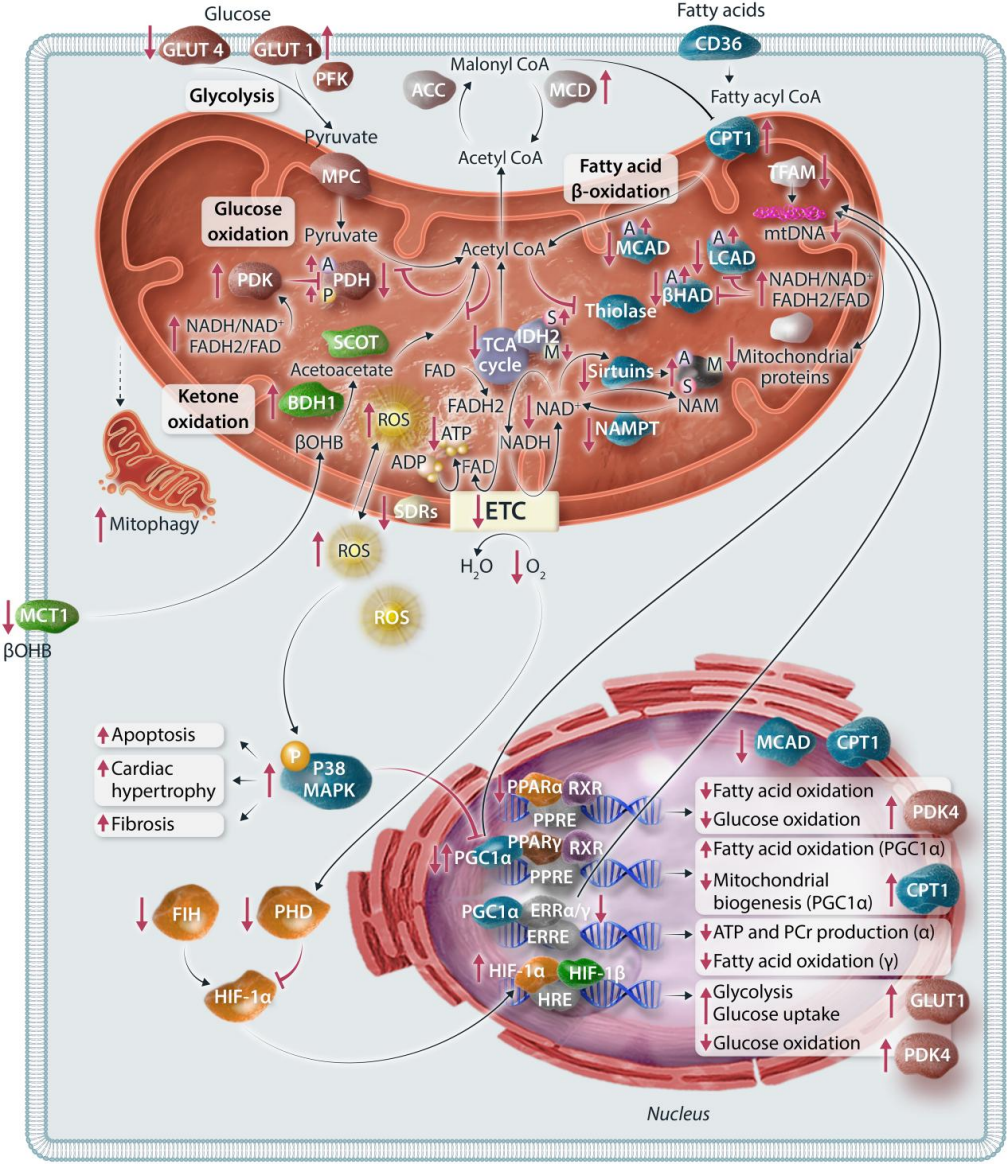
Molecular mechanisms contributing to altered energy metabolism in heart failure. Changes and disruptions in energy metabolism in heart failure are governed by four distinct molecular mechanisms: mitochondrial function, substrate control, transcriptional control, and post-translational modifications. (i) Mitochondrial dysfunction in heart failure encompasses reduced transcriptional regulation of mitochondrial biogenesis, increased ROS production, impaired mitofission, sustained mitophagy, and enhanced autophagic cell death of cardiomyocytes. (ii) Regulation of carbon flux through metabolic pathways in the heart relies on tight control of CoA and its derivatives. In heart failure, the acetyl-CoA pool resulting from fatty acid oxidation can allosterically inhibit metabolic pathways through enzymes like PDH, thiolase, and ACAT. (iii) Transcriptional regulation in cardiac metabolism involves PPARs, ERRs, PGC-1α, and HIF-1α. (iv) PTMs play a crucial role in modulating the activities of mitochondrial enzymes and are implicated in heart failure progression. The small bubbles attached to specific proteins with letters P, A, S, M are representing PTMs. The representation of each letter are phosphorylation, P; acetylation, A; succinylation, S; malonylation, M. GLUT1/4, glucose transporter 1/4; PFK, phosphofructokinase; MPC, mitochondrial pyruvate carrier; PDH, pyruvate dehydrogenase; PDK, PDH kinase; CD36/FAT, fatty acid transporter; CPT1, carnitine palmitoyltransferase 1; LCAD, long-chain acyl-CoA dehydrogenase; βHAD, 3-OH-acyl-CoA dehydrogenase; MCAD, medium chain acyl-CoA dehydrogenase; βOHB, β-hydroxybutyrate; BDH1, βOHB dehydrogenase 1; SCOT, succinyl-CoA:3-ketoacid CoA transferase; MCT1, monocarboxylate transporter 1; ROS, reactive oxygen species; TCA, tricarboxylic acid; NADH, nicotinamide adenine dinucleotide; FADH2, flavin adenine dinucleotide; ETC, electron transport chain; ADP, adenosine diphosphate; ATP, adenosine triphosphate; SDR, short chain dehydrogenase; IDH2, isocitrate dehydrogenase; NAMPT, nicotinamide phosphoribosyltransferase; NAM, nicotinamide; TFAM, transcription factor A, mitochondrial; PGC-1α, peroxisome proliferator-activated receptor-gamma coactivator (PGC)-1α; MAPK, mitogen-activated protein kinase; PPAR, peroxisome proliferator-activated receptors; RXR, the retinoid X receptor; ERR, oestrogen-related receptor; HIF1, hypoxia-induced factor-1; HRE, hypoxia-response element.

Mitochondrial function is compromised in the failing heart due a number of reasons, including: (i) reduced transcriptional regulation of mitochondrial biogenesis, (ii) increased reactive oxygen species (ROS) production, (iii) impairments in mitofission, (iv) sustained mitophagy, and (v) increased autophagic cell death of cardiomyocytes (*Figure 3*).^184–186^ Mitochondrial biogenesis is also altered in the failing heart. For instance, peroxisome proliferator-activated receptor-gamma coactivator-1α (PGC-1α), a member of a family of transcription coactivators that plays a central role in regulating mitochondrial biogenesis, is decreased in HFrEF.^93,187–189^ In addition, the expression of p38 mitogen-activated protein kinase (MAPK), which modulates PGC-1α activity, is reduced in failing rat hearts caused by MI.^190^ Mitochondrial DNA content and mitochondrial DNA-encoded proteins are also decreased in patients with heart failure.^191^ An increased abundance of PGC-1α accompanies these alterations in mitochondrial DNA levels, but there is a decreased expression of oestrogen-related receptor α (ERRα) and mitochondrial transcription factor A.^191^ These findings highlight the role of PGC-1α in modulating mitochondrial biogenesis signalling in advanced heart failure.

Increased levels of cardiac ROS have been implicated in mitochondrial damage in heart failure.^192,193^ MAPK activation, cardiac hypertrophy, and fibrosis have also been linked to angiotensin II-induced mitochondrial ROS in HFrEF mice.^194^ In a mouse model of hypertensive cardiomyopathy induced by angiotensin II infusion,^195^ mitochondria-targeted antioxidant (N-acetyl cysteine) treatment, but not non-targeted ROS scavenging, mitigated cardiomyopathy. These findings emphasize the role of mitochondrial ROS in triggering structural remodelling and mitochondrial defects in heart failure. However, attempts to reduce ROS generation and/or levels in heart failure using antioxidant interventions in human studies have not been overwhelmingly encouraging.^196,197^ Therefore, the effectiveness of these antioxidant interventions in inhibiting cardiac ROS in patients with heart failure and whether ROS generation is a possible therapeutic target to limit mitochondrial damage and cardiac remodelling in heart failure are still outstanding questions that need to be answered in future studies.

Mitochondrial fission and mitophagy are closely linked, with mitochondrial fission facilitating the segregation of dysfunctional components of the mitochondria into an organelle that can undergo mitophagy (degradation). Cardiac-specific deletion of proteins involved in mitofission (preventing the assembly of these organelles) leads to the development of heart failure *in vivo*.^185,186^ Interestingly, disrupting the mitofission process also leads to the accumulation of defective mitochondria and an increased mitophagy of the entire mitochondria instead of mitophagy restricted to organelles containing the dysfunctional mitochondrial components.^185,186^ The heart responds to pressure overload stresses by increasing mitophagy, which removes damaged mitochondria to maintain ATP production. However, in cases of sustained mitophagy like heart failure, excessive mitochondrial clearance can occur, thereby reducing the number of mitochondria in the heart. On the other hand, when mitophagy is impaired, dysfunctional mitochondria cannot be properly degraded and continue to cause damage, leading to autophagy of the cardiomyocyte over time.^186,198^

### 6.2 Alterations in substrate control

Carbon flux through different energy metabolic pathways highly depends on the overall levels of coenzyme A (CoA) and its derivatives (*Figure 3*). These energy-baring molecules have major roles in controlling metabolic flux through these various pathways at three different levels: (i) as substrate and products of the metabolic pathways, (ii) as allosteric regulators of the metabolic pathways, and (iii) as a regulator of fatty acid oxidation via malonyl-CoA. Cytosolic CoA levels are very low in heart muscle (15–50 μM),^199^ but they are required to esterify fatty acids to long-chain acyl-CoA.^200^ In contrast, mitochondrial CoA levels are higher (2 mM), and they are prerequisites for acetyl-CoA production from pyruvate decarboxylation by PDH and catabolism of ketone bodies and amino acids.^200,201^ Acetyl-CoA production rates from these metabolic pathways are closely matched to its entry into the TCA cycle, allowing cardiac ATP production to match the heart's energy demand.^202,203^ At rest or during low cardiac workload, mitochondrial acetyl-CoA/CoA is increased, which feeds back and allosterically inhibits fatty acid oxidation at the level of 3-ketoacyl-CoA thiolase (KAT).^202^ High acetyl-CoA/CoA ratio also inhibits glucose oxidation and ketone oxidation at the levels of PDH and mThiolase, respectively.^200,202^ The increase in acetyl-CoA/CoA ratio controls carbohydrate oxidation by direct inhibition of PDH and/or stimulation of PDK, which phosphorylates and inhibits PDH due to the increase in acetyl-CoA/CoA ratios. However, increased energy demand and workload decrease the acetyl-CoA/CoA ratio due to increased entry of acetyl-CoA into the TCA cycle.^199^ As a result, the feedback inhibition on KAT, PDH, and mThiolase decreases, increasing the oxidation of fatty acids, carbohydrates, and ketones, respectively.^199,201,202^ Studies have shown a significant decrease in cardiac acetyl-CoA/CoA ratio in HFrEF and the infarcted heart.^52,204^ This is mainly due to the overall reduction in mitochondrial oxidative metabolism and in acetyl-CoA generation through the different metabolic pathways.^52,90,204^

Mitochondrial fatty acid β-oxidation is regulated by cytosolic malonyl-CoA levels and their effect on CPT1.^36,205^ Malonyl-CoA inhibits CPT1 and decreases fatty acid uptake into the mitochondria by inhibiting the conversion of long-chain acyl-CoA to long-chain acylcarnitine, thereby inhibiting fatty acid β-oxidation. Malonyl-CoA levels are rapidly turned over,^206^ with its production controlled by ACC and its degradation by MCD. Therefore, ACC activity negatively correlates to fatty acid β-oxidation rates, while MCD activity positively correlates with fatty acid β-oxidation rates.^207–211^ For example, genetic deletion or pharmacological inhibition of MCD is associated with an increase malonyl-CoA levels and a decrease in cardiac fatty acid oxidation.^212,213^ In obesity and diabetes, cardiac fatty acid ß-oxidation rates increase, leading to an increase in acetyl-CoA generation.^163,204^ Impaired mitochondrial TCA cycle activity, such as during ischaemia and severe heart failure, can also increase mitochondrial acetyl-CoA levels.^52,204^ The mitochondrial acetyl-CoA production in these conditions may exceed the oxidative capacity of the TCA cycle and, therefore, increase acetyl-CoA levels. Augmented levels of fatty acids seen in these conditions also increase cardiac MCD expression, contributing to a decrease in malonyl-CoA levels.^214^

Another layer of allosteric regulation of the flux through different metabolic pathways is the levels of reduced equivalents, namely NADH and FADH_2_. NADH and FADH_2_ are mainly generated from the metabolism of fatty acids, glucose, ketone bodies, amino acids, and TCA intermediates. Reduced equivalents can also be generated cytosolic glycolysis and shuttled into the mitochondria via the malate-aspartate shuttle or the glycerol phosphate shuttle.^200^ Similar to the acetyl-CoA/CoA ratio, the ratios of NADH/NAD^+^ and FADH_2_/FAD are important regulatory mechanisms of the dehydrogenases involved in fatty acid β-oxidation, glucose oxidation, ketone oxidation, amino acid oxidation, and TCA cycle activity. Under low energy demand, the ratios of NADH/NAD^+^ and FADH_2_/FAD increase, that feeds back and slows down carbon flux through the different metabolic pathways. High NADH/NAD^+^ and/or FADH_2_/FAD ratios also allosterically trigger PDK activity, phosphorylating, and inhibiting PDH activity, thereby decreasing glucose and lactate oxidation.^215^ NAD^+^ availability is a crucial determinant for Sirtuin activity (as discussed in the next section). In myocardial ischaemia, there is a decrease in the NAD^+^/NADH ratio as a result of reduced ETC activity, which contributes to the inactivation of SIRT3 and leads to the hyperacetylation of mitochondrial proteins.^52,216^ It is important to note that during I/R injury, nicotinamide phosphoribosyltransferase (NAMPT), the rate-limiting enzyme that converts nicotinamide (NAM) to nicotinamide mononucleotide in the NAD^+^ salvage synthesis pathway, is down-regulated in the heart. Consequently, the NAD^+^ levels in the heart decrease and this results in a reduction in sirtuin activity.^217,218^ Studies have shown the cardioprotective effect of enhancing NAD^+^ levels in I/R injury either by exogenous NAD^+^ supplementation or enzymatic manipulation.^217,219,220^

### 6.3 Transcriptional regulation

Expressed in cardiomyocytes, peroxisome proliferator-activated receptors (PPARs) constitute a pivotal pathway regulating cardiac fatty acid uptake and oxidation at the transcriptional level (*Figure 3*). Within the heart, three forms of PPARs are present, namely the more prevalent PPAR-α and PPAR-β/δ, alongside PPAR-γ. Extensive research has focused on elucidating the role of PPAR-α in governing fatty acid metabolism in cardiac tissue. Studies employing gain/loss-of-function approaches in mice have underscored the significance of PPAR-α in modulating the expression of genes involved in most steps of cardiac fatty acid utilization, such as the expression of CPT1 and medium chain acyl-CoA dehydrogenase 1 (MCAD1).^221–223^ Nevertheless, understanding the regulatory mechanisms of PPAR-α in heart failure remains complex. Notably, elevated levels of PPAR-α have been associated with diabetes, with chronic activation of PPAR-α resulting in a diabetic-like cardiac phenotype characterized by heightened fatty acid utilization alongside reduced glucose oxidation.^165,180,224^ Additionally, increased levels of PPAR-α have been demonstrated to exacerbate cardiac recovery impairment following ischaemia/reperfusion injury *ex vivo*.^225^ Similarly, PPAR-α agonism in hypertrophic rat hearts diminishes cardiac power and efficiency.^226^ Conversely, Kaimoto *et al.*^227^ showed that early-stage activation of PPAR-α in heart failure improves cardiac function and enhances the expression of fatty acid oxidation genes in mice. To reconcile these disparate findings, studies have suggested that PPAR-α can function as both an activator and repressor of transcription and the down-regulation of PPAR-α in heart failure may primarily affect a subset of fatty acid oxidative genes rather than entail global down-regulation.^228,229^

As a transcriptional coactivator of PPAR-γ, gene expression analysis has revealed that PGC-1α targets pathways including fatty acid oxidation, mitochondrial biogenesis, and OXPHOS.^230–232^ Although PGC-1α has been implicated as a crucial pathway in metabolic deterioration and cardiac remodelling in the failing heart, evidenced by accelerated development of heart failure, deficits in cardiac energy reserve and function, as well as accelerated cardiac remodelling in PGC-1α KO mice,^189,233,234^ the regulation of PGC-1α expression is intricate and may strongly depend on the cause and timing of disease progression. Animal studies have reported decreases in PGC-1α expression in heart failure.^235–238^ Similarly, evidence from patients at an advanced-stage of heart failure has shown decreased gene or protein expression.^239,240^ However, both murine and human studies have not consistently found changes in PGC-1α gene expression in heart failure^191,241^; some even demonstrated an increase in expression. For example, in a mouse model of angiotensin II-induced hypertrophied heart, an increase in mRNA expression of PGC-1α was associated with a concurrent increase in mitochondrial turnover.^194^ Moreover, patients with advanced-stage heart failure have also exhibited slight increases in gene or protein expression, suggesting heightened fatty acid oxidation in the failing heart.^242^

ERR also play a crucial role in the transcriptional regulation of cardiac metabolism. This family comprises three members (ERR-α, ERR-β, and ERR-γ), with ERR-α and ERR-γ being the predominant isoforms in cardiac tissue.^243^ Studies have shown that by stimulating ERR-α on PPAR-α, these receptors facilitate fatty acid uptake and β-oxidation.^244^ In pressure overload-induced heart failure, the deletion of ERR-α has been linked to reduced maximal ATP production and depletion of phosphocreatine, underscoring its significance in the bioenergetics and adaptive functions of a stressed heart.^243^ Recent findings have also highlighted the potential of novel pan-ERR agonists in mitigating heart failure *in vivo*.^245^ Primarily activating ERR-γ, agonism up-regulates genes associated with fatty acid metabolism and mitochondrial function. Consequently, this up-regulation significantly improved EF, improved fibrosis, and enhanced survival in pressure overload-induced heart failure.

As a subunit of the heterodimeric transcription factor hypoxia-inducible factor 1 (HIF-1), the primary function of HIF-1α is to regulate oxygen (O_2_) levels by balancing O_2_ supply and demand.^246^ Under hypoxic conditions, the activity of HIF-α increases due to decreased activities of its regulators prolyl hydroxylase domain (PHD) and factor inhibiting HIF, caused by the up-regulation of mitochondrial α-ketoglutarate dehydrogenases.^247^ In heart failure, HIF-1α responds to cardiac stress and hypoxic states by promoting glycolysis to cope with reduced oxygen levels, as evidenced by its direct activation of GLUT1 to facilitate myocardial glucose uptake.^248,249^ However, the increased glucose uptake and utilization is not accompanied by a rise in pyruvate oxidation. Significantly, HIF-1α suppresses mitochondrial function and O_2_ consumption by up-regulating and activating PDK, therefore inhibiting PDH activity and mitochondrial pyruvate/glucose oxidation.^248^

### 6.4 Post-translational modifications

Through the addition or deletion of distinct carbon and non-carbon groups, post-translational modifications (PTMs) [including phosphorylation, acetylation, succinylation, malonylation, glutarylation, and O-GlcNAcylation (O-GlcNAc)] are instrumental in modifying the activities of mitochondrial enzymes, and alterations in these PTMs are implicated in the progression of heart failure. Phosphorylation has been extensively studied in the heart given its substantial role in modulating glucose and fatty acid metabolism.^52,84,204^ Recent attention has shifted towards investigating other PTMs in the context of heart failure (*Figure 3*).

Lysine acetylation is a highly regulated process involved in various metabolic changes observed in heart failure, diabetes, and obesity.^130^ This modification occurs through enzymatic and non-enzymatic pathways, wherein an acetyl group is transferred from acetyl-CoA to a lysine residue. Moreover, acetylation can be reversed by histone deacetylases (KDACs), encompassing two main families: Zn^2+^-dependent DACs (KDAC1–11) and NAD^+^ -dependent KDACs (sirtuins 1–7). Notably, SIRT3, the major mitochondrial deacetylase, acts as a crucial link between NAD^+^ homeostasis and changes in myocardial energetics by modulating enzymes involved in, but not limited to, glucose, fatty acid, and ketone metabolism.^218^ In heart failure, depletion of SIRT3 expression results in an overall hyperacetylated state, disrupting cardiac metabolism, and exacerbating disease progression.^145,250^ Besides a decrease in phosphorylation of PDH in heart failure due to hyperacetylation of PDH phosphatase,^251^ studies have demonstrated the inhibition of PDH activity through direct acetylation in the failing myocardium of mice, leading to an overall decrease in glucose metabolism.^252,253^ Similarly, SIRT3 also regulates fatty acid oxidation enzymes such as long-chain acyl-CoA dehydrogenase (LCAD) and 3-hydroxy acyl-CoA dehydrogenase (β-HAD). Interestingly, there is conflicting evidence regarding the role of acetylation in fatty acid oxidation in the failing heart. Alrob *et al.*^166^ showed that fatty acid oxidation rates are increased in SIRT3 KO hearts, associated with increased acetylation and activity of LCAD and β-HAD. Conversely, other studies found that heart failure associated with decreased SIRT3 expression or KO was linked to increased LCAD acetylation and impaired fatty acid oxidation, with the activation of SIRT3 mediating deacetylation of LCAD and promoting fatty acid oxidation.^254–256^ Recently, SIRT3 activity has also been associated with ketone metabolism and its corresponding protective effects in heart failure. SIRT3 KO in neonatal rat cardiomyocytes leads to reductions in the ketone metabolism enzyme (SCOT and MCT1) expression while abolishing the beneficial effects of choline on Ang-II-induced hypertrophy.^257^ Additionally, Yan *et al.* also demonstrated that decreased SIRT3 expression in HFpEF in mice is associated with decreases in ketone metabolism proteins, including BDH1, SCOT, and acetyl-CoA acetyltransferase 1 (ACAT1). They further found that SIRT3 KO in HFpEF mice subsequently worsened HFpEF phenotypes such as cardiac hypertrophy and myocardial fibrosis.^258^

Regulated by SIRT5, succinylation, malonylation, and glutarylation are lesser investigated lysine acylation modifications involved in mitochondrial energetics. Although little is known about the specific mitochondrial enzymes succinylation catalyzes, increased succinylation in SIRT5KO cardiac mitochondria has been shown to associate with increased 3-hydroxyacyl dehydrogenase flux, suggesting an increase in fatty acid oxidation.^259^ Additionally increased succinylation of fatty acid oxidation enzymes in newborn hearts is associated with enhanced activity of these enzymes.^260^ However, the precise impact of succinylation on heart failure metabolism remains unresolved. TAC-induced heart failure in mice is associated with inhibited SIRT5 expression in the mitochondria, increased isocitrate dehydrogenase 2 (IDH2) succinylation, and decreased cardiac function attributable to decreased mitochondrial energy metabolism.^261^ Interestingly, supplementation with quercetin, a flavonoid, increased SIRT5 expression and promoted IDH2 desuccinylation, concomitantly inhibiting myocardial fibrosis and improving cardiac function.^261^ Additionally, Takada *et al.*^262^ observed that the overall myocardial mitochondrial protein succinylation levels were significantly lower in MI mice. However, some TCA cycle enzymes and membrane proteins, such as IDH2, had higher succinylation levels, which decreased subsequent to additions of succinyl-CoA.^262^ Similar to succinylation, the significance of malonylation in cardiac energy metabolism and other cellular processes in heart failure is not yet fully understood. In the normal heart, lysine malonylation involves proteins associated with protein translation, amino acid catabolism, the TCA cycle, and ATP synthesis. It has also been demonstrated that enhanced malonylation is associated with greater PDH activity in MCD KO heart mitochondria, suggesting malonylation's role in promoting glucose oxidation.^259^ Furthermore, increased malonylation through inhibition of MCD activity stimulates glucose oxidation, thereby protecting the ischaemic heart.^213^ Recently, LC-MS-based label-free lysine malonylation profiling of mice hearts after TAC surgery identified 679 malonylated sites in 330 proteins.^263^ Specifically, a decrease in IDH2 malonylation was detected both *in vitro* and *in vivo*, potentially linking malonylation to cardiac hypertrophy. Lastly, while hyperglutarylation of proteins involved in fatty acid metabolism, aerobic metabolism, the TCA cycle, and coenzyme metabolism has been demonstrated,^264^ the significance of this type of modification in heart failure has yet to be determined.

## 7. Targeting cardiac energy metabolism to treat heart failure

### 7.1 Targeting fatty acid oxidation

As discussed, there is considerable confusion about what alterations in fatty acid oxidation occur in heart failure, with decreases,^88,99,108^ no changes,^81,82^ or increases^78^ in fatty acid oxidation reported in the failing heart.

**Figure 4:**
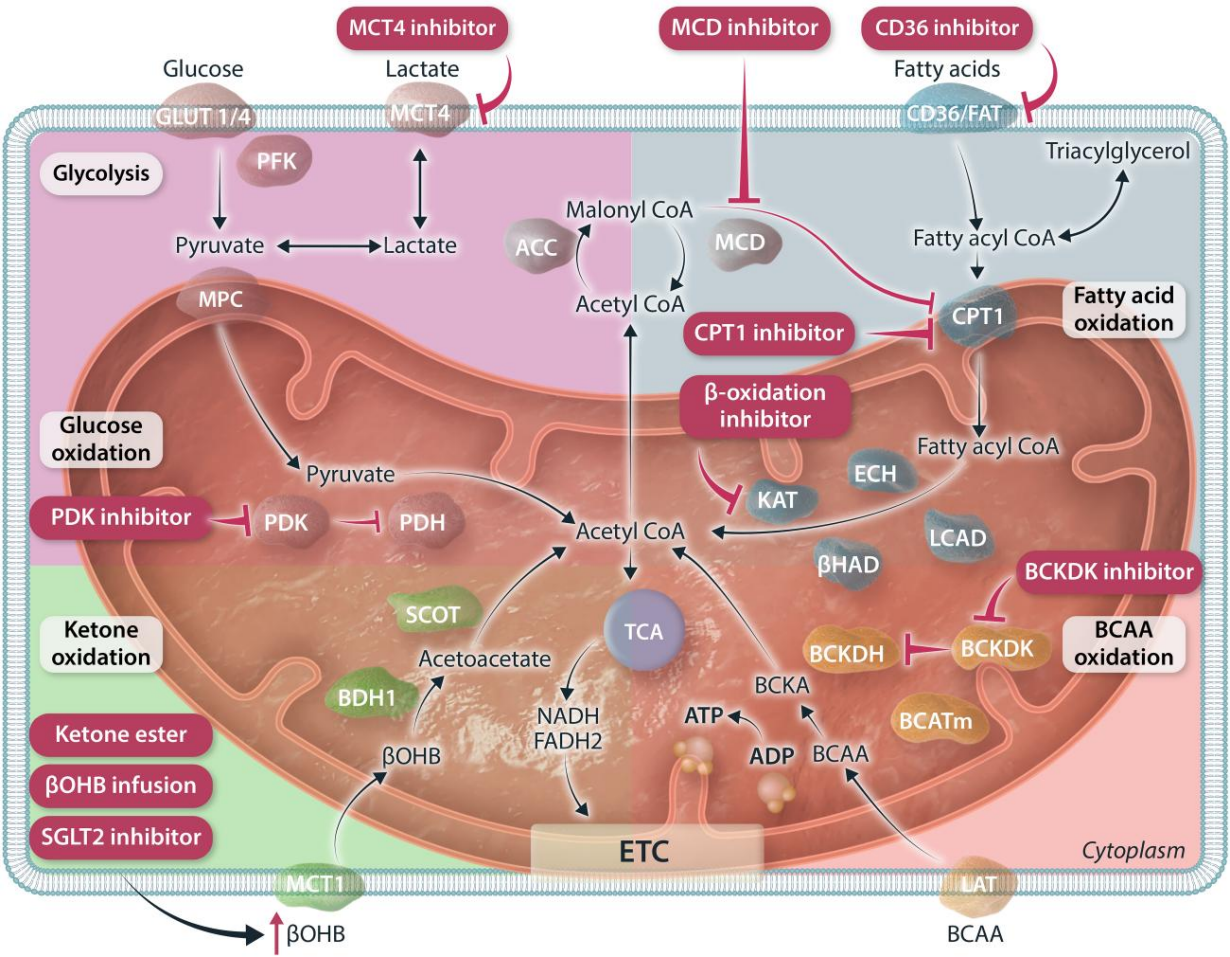
Current available pharmacological therapies that target cardiac energy metabolism. Collection of drugs that target different metabolic pathways through modifying the action of key enzymes involved. PDK inhibitor alleviates the inhibitory action of PDK on PDH, thus can stimulate glucose oxidation. MCT4 inhibition decreases lactate efflux, which can redirect glycolytic carbon flux into mitochondrial pyruvate oxidation/glucose oxidation. CD36 inhibition decrease fatty acid uptake into myocardium. Both CPT1 inhibitors and MCD inhibitors decrease the action of CPT1 for uptake of fatty acids into mitochondria. β-oxidation inhibitors interferes with the action of KAT, one of the major β-oxidation enzymes. Ketone therapy includes, ketone ester, βOHB infusion, SGLT2 inhibitor all which increases circulating βOHB levels, thus elevated accessibility of βOHB for further uptake and oxidation by the heart. BCKDK inhibitors alleviate the inhibitory action of BCKDK, thus stimulating the action of BCKDH and inducing the flux of BCAA oxidation. GLUT1/4, glucose transporter 1/4; MCT4, monocarboxylate transporter 4; PFK, phosphofructokinase; MPC, mitochondrial pyruvate carrier; PDH, pyruvate dehydrogenase; PDK, PDH kinase; ACC, acetyl-CoA carboxylase; MCD, malonyl-CoA decarboxylase; CD36/FAT, fatty acid transporter; CPT1, carnitine palmitoyltransferase 1; LCAD, long-chain acyl-CoA dehydrogenase; ECH, enoyl CoA hydratase; βHAD, 3-OH-acyl-CoA dehydrogenase; KAT, 3-ketoacyl-CoA thiolase; BCAA, branched-chain amino acids; BCATm, mitochondrial BCAA amimotransferase; BCKDH, branched-chain α-ketoacid dehydrogenase; BCKDK, BCKDH kinase; LAT, L-type amino acid transporters; βOHB, β-hydroxybutyrate; BDH1, βOHB dehydrogenase 1; SCOT, succinyl-CoA:3-ketoacid CoA transferase; SGLT2, sodium-glucose co-transporter 2; TCA, tricarboxylic acid; NADH; nicotinamide adenine dinucleotide; FADH2, flavin adenine dinucleotide; ETC, electron transport chain; ADP, adenosine diphosphate; ATP, adenosine triphosphate.

This has led to opposing studies aimed at either using pharmacological strategies to increase^265,266^ or decrease^267,268^ fatty acid oxidation in the failing heart. While no large clinical trials have yet been performed, currently avaliable clinical evidence does support the concept of inhibiting fatty acid oxidation to treat heart failure (*Figure 4*).^269–274^ Chronic treatment with trimetazidine, a partial inhibitor of fatty acid oxidation, improves functional class, quality of life, and left ventricular function in patients with HFrEF, regardless of its aetiology, and diabetic status.^269^ A meta-analysis also confirmed that trimetazidine has significant protective effects against all-cause mortality, cardiovascular events, and hospitalization in patients with heart failure.^271^ These beneficial effects of trimetazidine are thought to be due to increased cardiac glucose oxidation, secondary to fatty acid oxidation inhibition, resulting in an increase in cardiac efficiency.^275^ Clinical studies with the fatty acid oxidation inhibitors, perhexiline and etomoxir, have also shown decreased heart failure severity in HFrEF patients.^270,272,273^ Although cardiac fatty acid oxidation rates are increased in HFpEF,^121^ no clinical data are available on whether fatty acid oxidation inhibition benefits these patients.

Ranolazine is approved in the USA and Europe as a second-line agent in the management of chronic coronary syndromes. It not only blocks sodium channels (I_Na_) but also inhibits cardiac fatty acid oxidation.^276^ A meta-analysis of eight clinical trials in HFpEF patients showed that ranolazine has good efficacy in improving diastolic performance, while not affecting blood pressure, heart rate, or rate of ventricular repolarisation (shortening of the QT interval).^277^ However, it remains unclear how much of this benefit originates from inhibition of cardiac fatty acid oxidation vs. INa inhibition.

At an experimental level, inhibiting cardiac fatty acid oxidation using a MCD inhibitor can improve heart function in rats subjected to heart failure due to permanent left anterior descending artery occlusion.^97^ In contrast, inhibition of ACC, which lowers malonyl-CoA and stimulates fatty acid oxidation, is cardioprotective in failing mice hearts due to pressure overload.^265,266^ While these beneficial effects of MCD and ACC inhibition may seem contradictory, both approaches result in an improved coupling of glycolysis to glucose oxidation, suggesting a decrease in proton production from uncoupled glucose metabolism. An alternate possible mechanism is inhibiting fatty acid oxidation in cardiomyocytes (due to CPT1ß deficiency), which has been proposed to improve resistance to hypoxia and to stimulate cardiomyocyte proliferation, allowing heart regeneration after ischaemia–reperfusion injury.^268^

Numerous mouse models have been produced in which myocardial fatty acid uptake or oxidation is either decreased or increased. This includes mice fed a high-fat diet, lipoprotein lipase KO mice, MCD KO mice, ACC KO mice, CPT1ß KO mice, CD36 KO mice, FATP1 overexpressing mice, acyl-CoA synthetase 1 overexpressing mice, diglyceride acyltransferase 1 (DGAT1) overexpressing mice, adipose triglyceride lipase (ATGL) overexpressing mice, ATGL KO mice, LCAD KO mice, PPARα KO mice, PPARα overexpressing mice, PGC1-α overexpressing mice, and PGC1α KO mice. In these mice there is no consensus on whether inhibiting or stimulating fatty acid oxidation is beneficial or harmful for either ischaemic or non-ischaemic heart failure (see Abudurrachim *et al.*^278^ for review). In general, if fatty acid oxidation is accompanied by increased glucose oxidation, then beneficial effects on cardiac function occur. However, contractile dysfunction occurs if fatty acid oxidation inhibition causes a cardiac energic deficiency or lipid accumulation.

A potential problem with mitochondrial fatty acid oxidation inhibition is that decreasing cardiac fatty acid oxidation with maintained fatty acid uptake has the potential to contribute to lipotoxicity.^279^ Inhibition of sarcolemmal fatty acid uptake by inhibiting FAT/CD36 is one potential approach to overcoming this issue. In support of this, deletion of cardiac FAT/CD36 reduces lipid accumulation and improves contractile function in mice prone to lipotoxic cardiomyopathy.^280^ The FAT/CD36 inhibitor sulfo-*N*-succinimidyl oleate also decreases fatty acid oxidation, decreases triacylglycerol accumulation, and improves cardiac function in diabetic rat hearts subjected to hypoxia-reoxygenation.^280^ Additionally, it is important to note that, even though pharmacological modulation of fatty acid metabolism has shown beneficial effects in improving cardiac function, but the results are not always consistent.^270^ As such, there should be a note of caution that the evidence has not been sufficient for the drugs to be adopted in international clinical practice guidelines.

### 7.2 Targeting glucose oxidation

Although glycolysis increases in heart failure, the subsequent oxidation of glycolytically derived pyruvate (glucose oxidation) is impaired.^6^ This results in an uncoupling of glycolysis from glucose oxidation, increasing proton production, and decreasing cardiac efficiency.^6^ Two potential therapeutic approaches to overcome this problem are to increase mitochondrial pyruvate uptake or to stimulate PDH (the rate-limiting enzyme for glucose oxidation) (*Figure 4*).

In heart failure patients, expression of the MPC is decreased, while the lactate exporter monocarboxylate transporter 4 (MCT4) is increased (which transports lactate-derived from glycolytically derived pyruvate that is not oxidized out of the cardiomyocyte). An inhibitor of MCT4 (VB124) has been developed that inhibits lactate efflux, which is thought to redirect glycolytic carbon flux into mitochondrial pyruvate oxidation.^102^ In support of this concept, treating cardiomyocytes with VB124 decreases their response to hypertrophic stress and increases mitochondrial pyruvate uptake.^102^ However, whether MCT4 inhibition can improve contractile function in either clinical or experimental heart failure has yet to be determined. Similarly, while a decrease in MPC function contributes to cardiac dysfunction,^101,102^ it is not yet known if stimulation of MPC can benefit the failing heart.

One approach to increasing glucose oxidation is to inhibit PDK, which normally phosphorylates and inhibits PDH. The most widely studied approach to do this is with DCA. DCA administration to patients with angina and coronary artery disease augments left ventricle stroke volume and enhances myocardial efficiency.^281^ DCA also improves left ventricle mechanical efficiency and enhances cardiac work in patients with New York Heart Association Classes III and IV congestive heart failure.^282^ Unfortunately, the potency and pharmacokinetics of DCA are not optimal for chronic treatment of heart failure patients. However, the concept of stimulating glucose oxidation with DCA to treat heart failure has been shown in a number of experimental studies. DCA improves function in hypertrophied rat hearts^75^ and post-ischaemic cardiac function and efficiency in rat hearts by stimulating glucose oxidation and improving the coupling between glycolysis and glucose oxidation.^283^ In hypertrophic rat hearts (induced by hyperthyroidism), DCA treatment significantly reduces the severity of hypertrophy and maintains cardiac output.^284^ Using hyperpolarized [^13^C] pyruvate and magnetic resonance imaging, DCA reverses the impaired glucose oxidation seen in the hyperthyroid heart.^284^ In a porcine model of volume overload heart failure, using magnetic resonance spectroscopy with hyperpolarized [^13^C] pyruvate it was found that DCA treatment can improve contractile reserve and decrease hypertrophy by augmenting carbohydrate metabolism.^285^ In HFpEF, myocardial glucose oxidation rates are also markedly depressed.^121^ We recently demonstrated that treatment of heart failure in mice with a PDK inhibitor (MMR-0013) increases cardiac glucose oxidation and improves survival and cardiac function.^151^ Combined, these data support the concept that increasing glucose oxidation, by inhibiting PDK is a potential promising approach to treating heart failure.

Glucose oxidation studies have shown an accumulation of pyruvate and other glycolytic intermediates in myocardial biopsies of patients with end-stage heart failure.^92,102,104,105^ The accumulation of these metabolites was accompanied by a reduction in the expression levels of the MPC and an increase in the phosphorylation of PDH.^92,102,104,105^ In line with these studies, direct measurement of glucose oxidation rates revealed that these rates are reduced in the failing heart.^52,204^ The expression of MPC is decreased in human and animal heart failure,^286,287^ which might contribute to the overall reduction in cardiac glucose oxidation in heart failure, a reproducible signature of the failing heart. The decrease in mitochondrial uptake and oxidation of pyruvate contribute to ‘energy starvation’ in heart failure.

Inhibition of mitochondrial pyruvate uptake by MPC subunits (MPC1 or MPC2) deletion or pharmacological inhibitors induces pathologic cardiac hypertrophy and heart failure.^101,102,286,287^ These functional and structural changes are associated with increased flux into non-oxidative pathways such as the HBP, glycogen synthesis, the PPP, and amino acid biosynthetic pathways. Consistent with this, overexpression of MPC in hearts subjected to pressure overload or treated isoproterenol and an inhibitor of the lactate transporter MCT4, which increased the generation of pyruvate and flux through the MPC, limits the flux through glucose non-oxidative metabolic pathways and decreases the severity of adverse remodelling.^102,286^ MPC deletion markedly reduces glucose oxidation and causes acceleration of both glycolysis and fatty acid oxidation to compensate for the reduction in glucose contribution to cardiac ATP production.^101^ Ketogenic diet (high-fat/low-carbohydrate diet) feeding mitigates adverse remodelling by further increasing fatty acid oxidation and blunting glycolysis, lactate and pyruvate accumulation and flux of glucose carbons into non-oxidative metabolic pathways such as the HBP and glycogen in MPC KO heart.^101^ However, a ketogenic diet feeding exacerbates cardiac insulin resistance and does not improve cardiac function in a mouse model of MI.^288^

### 7.3 Targeting ketone oxidation

Increasing cardiac ketone oxidation has recently attracted attention as a potential therapeutic strategy to treat heart failure (*Figure 4*).^289^ In HFrEF, cardiac ketone oxidation rates are elevated, which has been proposed as an adaptive mechanism to compensate for the impaired overall myocardial energy production.^90,124^ Although the benefits of increasing cardiac ketone oxidation have been suggested to ketones being a thrifty fuel,^85,86,290^ our studies do not support this,^90^ and rather suggest that increasing ketone supply to the heart provides the failing heart with an extra fuel source and helps to improve overall cardiac energetics. In HFpEF, cardiac ketone oxidation rates are not increased, but can be decreased.^121,145^ This may contribute to an energy deficit in the heart. Several strategies have been evaluated to increase ketone supply to the heart, including direct ketone infusions, ketone ester supplementation, administering a ketogenetic diet, and using SGLT2 inhibitor treatment.^128^ Most of these increasing ketone supply strategies were tested in pre-clinical and clinical studies only for HFrEF.

Acute infusions of the ketone βOHB into HFrEF patients improves haemodynamic function, as evidenced by an increase in %EF.^70^ But the decreased cardiac efficiency in HFrEF patients was not improved with βOHB infusions, supporting the concept that ketones are not a more efficient fuel for the heart. In canines subjected to HFrEF using a chronic cardiac pacing protocol, the direct infusion of the βOHB ameliorates pathological cardiac remodelling.^126^ This infusion of βOHB elicits changes in the myocardial metabolic profile, with a significant increase in ketone body uptake and a decrease in glucose uptake.^126^ This suggests that ketones might compete with other myocardial substrates such as glucose. On the contrary, another study found that increasing βOHB supply to the heart does not decrease glucose oxidation or glycolysis rates in mice subjected to TAC-induced heart failure mice.^90^ Regardless, further studies are needed to ascertain the benefits of ketones in heart failure and to investigate how ketone oxidation fits into the overall bigger picture of myocardial energy metabolism in heart failure.^291^

A ketogenic diet and ketone ester supplements can also be used to increase circulating ketone concentrations. Ketogenic diets are characterized as low-carbohydrate high-fat diets that induces the body to undergo a ‘pseudo-fasted’ state,^292,293^ which stimulates the liver to use free fatty acids to produce ketone bodies, known as the process of ketogenesis.^294,295^ The liver is the main organ for ketogenesis, although some recent studies also suggest that the heart is capable of ketogenesis.^296,297^ Although the ketogenic diet has been widely used as an effective weight loss strategy, its applicability in patients with heart disease is limited. In healthy individuals consuming the ketogenic diet, there is evidence of elevated PCr/ATP ratios.^298^ However, whether this would induce comparable results in patients with heart failure remains unclear. The results gathered to date are rather conflicting, with some studies showing improved cardiac function in pre-clinical mice models of heart failure receiving ketogenic diets,^299,300^ while others showing adverse effects with continuous ketogenic diet feeding.^301^ In support of this, a study found that increasing βOHB through ketogenic diet can lead to decreased mitochondrial biogenesis, increased cardiomyocyte apoptosis, and the development of cardiac fibrosis.^302^ Guo *et al.*^301^ proposed that an alternate-day ketogenic diet can improve the effects of the ketogenic diet, while maintaining its protective effects. Additionally, supplementing more medium chain triglyceride within the ketogenic diets was shown to recover an age-related decrease in succinate dehydrogenase activity and coverage of metabolically active mitochondria within the myocardium.^303^ We recently demonstrated that a ketogenic diet is not cardioprotective in a post-MI mouse model of heart failure.^288^ This appeared to be due to a marked increase in myocardial fatty acid oxidation and decrease in glucose oxidation seen in mice on the ketogenic diet. Considering that ketogenic diet has a high-fat content, more research is required to ascertain the safety and feasibility of the ketogenic diet in usage for patients with heart failure.

Administration of ketone esters is another approach to increase circulating ketone levels. Ketone ester is palatable by oral administration, as such, would be more convenient to ingest compared to direct infusion with ketone bodies. Ketone esters are precursors of ketones and will be converted to βOHB. Two examples of ketone esters are R-1,3-butanediol and ethyl (R)-3-hydroxybutyrate, which can increase blood ketone levels up to 6 mM in humans.^304,305^ Chronic supplementation with ketone ester has demonstrated cardioprotective effects against MI-induced heart failure in both mice and rats.^306^ Around the same time, another study published the results showing that acute ketone ester intake can improve cardiac function in healthy participants.^307^ This suggests the potential to use ketone ester in treating a wide range of cardiovascular diseases given its ease of administration, relatively safe profile, and strong cardioprotective echocardiographic and haemodynamic effects. That being said, to this day, there is no human study that shows cardioprotective effects with the use of ketone ester in patients with heart failure. Some of the on-going and/or completed yet not published clinical trials include: NCT04442555, which investigate the haemodynamic effect of exogenous ketones in acute heart failure and cardiogenic shock; NCT04633460, which assessed the effect of ketone ester, DeltaG, on exercise performance in people with heart failure; NCT05159570, which investigate the effect of 14 days ketone ester supplementation on whole body and skeletal metabolism; NCT06108076, which evaluates both acute and chronic effects of oral ketone monoester in subjects with HFrEF and diabetes; NCT06078683, which studies the effects of acute and chronic ketone ester consumption on exercise tolerance and cardiac function in subjects with the metabolic phenotype of HFpEF; NCT05348460, which assess effects of a single oral dose of ketone ester on exercise performance in patients with chronic heart failure; and NCT04698005, which investigates the effects of ketone monoester in acutely decompensated heart failure. The results from these clinical trials will offer valuable information for guiding the potential usage and application of ketone ester as a potential therapeutic strategy for heart failure.

Another approach to increase circulating ketones in heart failure patients is with the used of SGLT2 inhibitors. SGLT2 inhibitors were first developed as anti-diabetic drugs^308–310^ but have recently been shown to be effective in treating heart failure.^152,309^ In the large-scale clinical trial, EMPA-REG OUTCOME, the SGLT2 inhibitor empagliflozin decreased both the rate of hospitalization and death due to cardiovascular outcomes in patients with T2D.^308^ The SGLT2 inhibitors dapagliflozin and canagliflozin can also reduce heart failure severity and incidence rates of cardiovascular hospitalization in patients with HFrEF both with and without diabetes, suggesting that the effects of SGLT2 inhibitor lie beyond its glucose-lowering effects.^309^ SGLT2 inhibitors have also been shown to benefit patients with HFpEF.^152^ The mechanisms by which SGLT2 inhibitors exert their cardioprotective effects are still unclear.^311^ The primary action of SGLT2 inhibitor is to decrease glucose reabsorption from the proximal tubule of the kidney.^312^ This can elicit a ‘pseudo-fasting’ state and induce ketogenesis by the liver. This can increase the ketone supply to the heart, fuelling the energy-starved/failure in midst of plenty failing heart.^313^ However, this may not be the case in HFpEF, as myocardial ATP production is not compromised in HFpEF mice.^121^ As such, SGLT2 inhibitors may also act through other pathways to improve cardiac function in HFpEF. In addition to ketone acting as an extra source of fuel for the heart, ketones can also act as a signalling molecule, exert anti-inflammatory effects and ameliorate oxidative stress.^127,314^ In particular, activation of NLRP3 inflammasome has been shown to be suppressed with βOHB, as well as the lowering of NLRP3 inflammasome-mediated production of IL-1β and IL-18.^314^ In addition, cardiac-specific overexpression of BDH1 in mice showed reduced oxidative stress in response to TAC-induced heart failure.^127^ As such, ketones likely have several pleiotropic roles in the setting of heart failure to exert its beneficial effects.

Although most studies aimed at increasing circulating ketones have been promising, some studies have raised concerns that chronic ketone exposure may impair cardiac insulin sensitivity through perturbation in the PI3K/PKB signalling cascade.^315,316^ Therefore, more studies are required to delineate the proper dosage and duration of ketone therapy for heart failure treatment.

### 7.4 Targeting BCAA oxidation

Impaired cardiac BCAA oxidation in HFrEF increases cardiac levels of BCAAs and BCKAs, which have been linked to adverse effects on heart function.^317–319^ Since the contribution of BCAA oxidation to ATP production in the heart is minimal compared to their oxidative substrates (glucose, fatty acid, and ketone bodies), it seems unlikely the detrimental effects associated with increased levels of BCAAs and BCKAs are mediated by increased BCAA contribution to cardiac ATP production. Instead, it seems likely that cardiac BCAAs and BCKAs have more important roles as signalling molecules to mediate the observed detrimental effects.^129^ Studies have shown that reducing the accumulation of cardiac BCAAs and BCKAs is a cardioprotective approach in different models of heart failure (*Figure 4*).^52,131,135,320,321^ This increase in BCAAs and BCKAs is likely due to downstream inhibition of BCKDH in the failing heart.^49,129,131,135,320,322^ Pharmacological enhancement of BCAA oxidation by stimulating BCKDH activity with BT2 (3,6-dichlorobenzo[b]thiophene-2-carboxylic acid, an inhibitor of BCKDK) treatment mitigates contractile dysfunction in pressure overload-induced heart failure mouse models.^131,135^ BT2 treatment also accelerates cardiac BCAA oxidation^49,135^ and improves post-MI cardiac function in a mouse model of myocardial ischaemia.^320^ Similarly, accelerating BCAA oxidation reduces infarct size in a mouse model of ischaemia/reperfusion injury.^321^ Of interest is that a recent study suggested that systemic activation of BCAA oxidation contributes to the cardioprotective effects of BT2.^134^ Systemic activation of BCAA oxidation is associated with reduced blood pressure in a mouse model of MI.^134^ However, it is unknown how BCAA modulates blood pressure and whether the effect of BCAA on blood pressure is specific to this mouse model of MI.

Another approach to decreasing cardiac BCAA and BCKA levels is by reducing whole-body BCAA intake. Interestingly, restricting BCAA levels in the diet stimulates cardiac fatty acid contribution to ATP production and limits cardiac triacylglycerol levels in Zucker fatty rats.^323^ Accumulation of cardiac BCAA levels has also been reported in patients with HFpEF.^143^ Similar to HFrEF, cardiac insulin resistance is also present in HFpEF, and it negatively influences cardiac energy metabolism and function in a mouse model of HFpEF.^121,151,324^ However, it is unknown whether cardiac BCAA oxidation is altered in HFpEF or whether stimulating cardiac BCAA oxidation influences cardiac insulin resistance and function in HFpEF. Taken together, these studies demonstrate that perturbations in cardiac BCAA oxidation and accumulation of BCAAs and BCKAs are strongly linked to the occurrence of cardiac insulin resistance, cardiac malfunction, and adverse remodelling in the failing heart.

### 7.5 Targeting non-glucose oxidation fates of glucose

In addition to being used for glycolysis and glucose oxidation, glucose taken up by the heart can also be directed to a number of other metabolic pathways. These include the polyol pathway, the HBP, the PPP, or the one-carbon cycle pathway.^101,105,286,287^

Aldose reductase (AR) is an important enzyme in the polyol pathway that converts glucose to sorbitol and mediates a number of pathways that exacerbate contractile dysfunction and adverse remodelling of heart failure.^325,326^ Studies have shown that the expression of AR in the heart also increases following ischaemia/reperfusion (I/R) injury, and this enhanced activity of cardiac AR has been linked to worsening post-ischaemic cardiac recovery.^326,327^ Consistent with this, whole body and cardiac-specific overexpression of AR in mice exacerbates post-ischaemic cardiac recovery following I/R injury.^326,327^ We have recently demonstrated that pharmacological inhibition of AR activity lessens diastolic dysfunction in a mouse model of diabetic cardiomyopathy.^328^ Interestingly, we found that this cardioprotective effect of AR inhibition was not associated with modifying glycolysis or glucose oxidation. Instead, AR inhibition inhibits cardiac fatty acid oxidation rates and mitigates cardiac fibrosis in mice with diabetic cardiomyopathy.^328^

The regulation of the cellular redox environment is closely linked to substrate metabolism, mainly through the PPP, a significant source of NADPH. NADPH plays a crucial role in proliferation and survival signalling.^329^ Additionally, NADPH maintains the pool of reduced antioxidants such as glutathione, which is a crucial defence against oxidative stress.^329^ Thus, the PPP may perform a dual role in regulating redox homeostasis. While much research has been conducted to study glycolysis and glucose oxidation, little is known about regulating *f* the PPP in heart failure. In dogs with pacing-induced heart failure, increased superoxide levels were attributed to the heightened activity of G6P dehydrogenase, which is the key oxidative enzyme of the PPP.^330^ If superoxide's effects are damaging, this activation of the PPP in the failing heart could be considered detrimental. However, G6P dehydrogenase-deficient mice develop higher oxidative stress and worsened contractile function after MI or aortic constriction.^331^ In Dahl salt-sensitive rats, Kato *et al.* found that flux through the PPP progressively increased during the development of heart failure. Significantly, they demonstrated that DCA treatment further increased this flux, which was associated with improved cardiac function.^75^ Taken together, the PPP is activated in heart failure. Although superoxide production may increase, currently available data support the notion that higher flux through the PPP in HF may represent a compensatory mechanism whose further activation could be of therapeutic relevance.

O-GlcNAc is one of the post-translational modifications that add the sugar moiety, β-D-*N*-acetylglucosamine, to a serine or a threonine residue along the peptide chain. Such PTM is uncharged, but the added sugar moiety is five times larger than the size of a phosphate group. The substrate for O-GlcNAc, β-*N*-acetylglucosamine, is produced through the HBP, which consumes ∼5% of intracellular glucose. Following glucose uptake, it is converted to fructose-6-phosphate (F6P) by hexokinase and isomerase. F6P is then converted to uridine diphosphate-*N*-acetylglucosamine (UDP-GlcNAc) in four enzymatic reactions. Among all the steps, glutamine:fructose-6-phosphate amidotransferase (GFAT) is the first and rate-limiting enzyme of the HBP. As for the enzyme that carries out the actual transfer of β-*N*-acetylglucosamine to the target protein, it would be the O-GlcNAc transferase. Wu *et al.*^332^ showed that salidroside administration is cardioprotective against I/R injury and improves cardiomyocyte glucose uptake by 1.7-fold, which was accompanied by a 1.6-fold increase in O-GlcNAc levels. Besides affecting glucose metabolism, fatty acid oxidation was also suggested to be under the impact of O-GlcNAc modification. Isolated rat hearts perfused with glucosamine (0–10 mM) showed an increase in O-GlcNAc levels and an increase in palmitate oxidation.^333^ This could be, at least in part, mediated by higher plasma membrane levels of the fatty acid transporter CD36.^334^ Interestingly, Li *et al.* demonstrated that chronic accumulation of BCAAs inhibits the HBP, thereby suppressing O-GlcNAc modification on cardiac proteins and sensitizing the heart to I/R injury.^321^ Excessive O-GlcNAc has also been linked to the development of cardiomyopathy, and attenuation of O-GlcNAc decreases the severity of pressure overload-induced contractile dysfunction and adverse cardiac remodelling.^335^ However, the data are not consistent regarding whether O-GlcNAc is beneficial or detrimental to the heart. For example, Kronlage *et al.*^336^ demonstrated that O-GlcNAc of HDAC4 is cardioprotective in diabetes mellitus and offsets pathological Ca^2+^/calmodulin-dependent protein kinase II signalling. Therefore, the effect of O-GlcNAc on cardiac function and structure needs further investigation.

## 8. Conclusions

In HFrEF, HFpEF, and heart failure associated with obesity and T2D, the energy metabolic profile of the heart undergoes dramatic changes, contributing to contractile dysfunction. The main metabolic change in all these forms of heart failure is a decrease in glucose oxidation. In HFpEF, diabetes and obesity, this decrease in glucose oxidation is accompanied by an increase in fatty acid oxidation, contributing to a decrease in cardiac efficiency (cardiac work/O_2_ consumed). Ketone oxidation is increased in HFrEF, which may be an adaptive process to provide the energy-starved failing heart with an extra fuel source. However, in HFpEF, diabetes, and obesity, ketone oxidation is decreased, contributing to a decrease in metabolic flexibility. These energy metabolic changes observed in the failing heart can be attributed to altered fuel availability, altered expression, altered allosteric control, and altered PTMs of key enzymes involved in various metabolic pathways. Targeting cardiac energy metabolism and/or optimizing the metabolic profile of the heart is a promising therapeutic strategy for treating heart failure. This includes stimulating glucose oxidation, stimulating ketone oxidation, or inhibiting fatty acid oxidation as an approach to increase metabolic flexibility and cardiac efficiency in the failing heart.

## Data Availability

No new data were generated or analyzed in this review article.
